# Development of a Quality Management Model and Self-assessment Questionnaire for Hybrid Health Care: Concept Mapping Study

**DOI:** 10.2196/38683

**Published:** 2022-07-07

**Authors:** Rosian Tossaint-Schoenmakers, Marise J Kasteleyn, Anneloek Rauwerdink, Niels Chavannes, Sofie Willems, Esther P W A Talboom-Kamp

**Affiliations:** 1 Saltro Diagnostic Centre, Unilabs Netherlands Utrecht Netherlands; 2 National eHealth Living Lab Leiden University Medical Centre Leiden Netherlands; 3 Department of Public Health and Primary Care Leiden University Medical Centre Leiden Netherlands; 4 Department of Surgery Gastroenterology and Metabolism Amsterdam University Medical Centre Amsterdam Netherlands; 5 Unilabs Group Geneve Switzerland

**Keywords:** quality assessment, hybrid health care, blended health care, eHealth, digital health, structure, process, outcome, concept mapping

## Abstract

**Background:**

Working with eHealth requires health care organizations to make structural changes in the way they work. Organizational structure and process must be adjusted to provide high-quality care. This study is a follow-up study of a systematic literature review on optimally organizing hybrid health care (eHealth and face to face) using the Donabedian Structure-Process-Outcome (SPO) framework to translate the findings into a modus operandi for health care organizations.

**Objective:**

This study aimed to develop an SPO-based quality assessment model for organizing hybrid health care using an accompanying self-assessment questionnaire. Health care organizations can use this model and a questionnaire to manage and improve their hybrid health care.

**Methods:**

Concept mapping was used to enrich and validate evidence-based knowledge from a literature review using practice-based knowledge from experts. First, brainstorming was conducted. The participants listed all the factors that contributed to the effective organization of hybrid health care and the associated outcomes. Data from the brainstorming phase were combined with data from the literature study, and duplicates were removed. Next, the participants rated the factors on importance and measurability and grouped them into clusters. Finally, using multivariate statistical analysis (multidimensional scaling and hierarchical cluster analysis) and group interpretation, an SPO-based quality management model and an accompanying questionnaire were constructed.

**Results:**

All participants (n=39) were familiar with eHealth and were health care professionals, managers, researchers, patients, or eHealth suppliers. The brainstorming and literature review resulted in a list of 314 factors. After removing the duplicates, 78 factors remained. Using multivariate statistical analyses and group interpretations, a quality management model and questionnaire incorporating 8 clusters and 33 factors were developed. The 8 clusters included the following: Vision, strategy, and organization; Quality information technology infrastructure and systems; Quality eHealth application; Providing support to health care professionals; Skills, knowledge, and attitude of health care professionals; Attentiveness to the patient; Patient outcomes; and Learning system. The SPO categories were positioned as overarching themes to emphasize the interrelations between the clusters. Finally, a proposal was made to use the self-assessment questionnaire in practice, allowing measurement of the quality of each factor.

**Conclusions:**

The quality of hybrid care is determined by organizational, technological, process, and personal factors. The 33 most important factors were clustered in a quality management model and self-assessment questionnaire called the Hybrid Health Care Quality Assessment. The model visualizes the interrelations between the factors. Using a questionnaire, each factor can be assessed to determine how effectively it is organized and developed over time. Health care organizations can use the Hybrid Health Care Quality Assessment to identify improvement opportunities for solid and sustainable hybrid health care.

## Introduction

### Background

In recent years, the use of eHealth has expanded, encouraged by the increasing pressure on health care [1,2] and growing interest in patient empowerment [3,4]. On the one hand, an aging population and an increase in chronic diseases are causing a higher and more complex demand for health care. In addition, the COVID-19 pandemic has accelerated pressure on health care [5-8]. Therefore, innovations such as eHealth are required to maintain accessibility and high quality of health care [9-12]. On the other hand, digital health technologies have significantly accelerated patients’ involvement [13-16]. In line with these developments, health care organizations have intensively integrated eHealth into traditional face-to-face consultations [17]. The combination of eHealth and face-to-face consultations can be defined as hybrid health care [18,19]. A few examples of hybrid health care are telemonitoring systems for patients with chronic diseases [20,21], web-based video coaching [22,23], and direct web-based access to medical records of patients [24,25], all of which are integrated into traditional health care.

Although health care organizations are increasingly providing hybrid health care, integrating eHealth into the daily care process is challenging. Working with hybrid health care requires organizations to change the way they work. The roles of health care providers and patients are changing, and the available resources are used differently [4,22,26,27]. Organizational structure and work processes must be adapted to ensure high-quality hybrid care [28-31]. Several studies have examined ways to promote eHealth adoption, such as increasing the adaptability of the technology or stakeholders’ value [32,33]. However, it remains challenging to organize hybrid health care effectively and sustainably [17]. There is a need for further research on how hybrid health care can be improved to add value to patients and health care providers when they work with eHealth. Therefore, we recently performed a systematic literature review to optimally organize hybrid health care [17].

**Figure 1:**
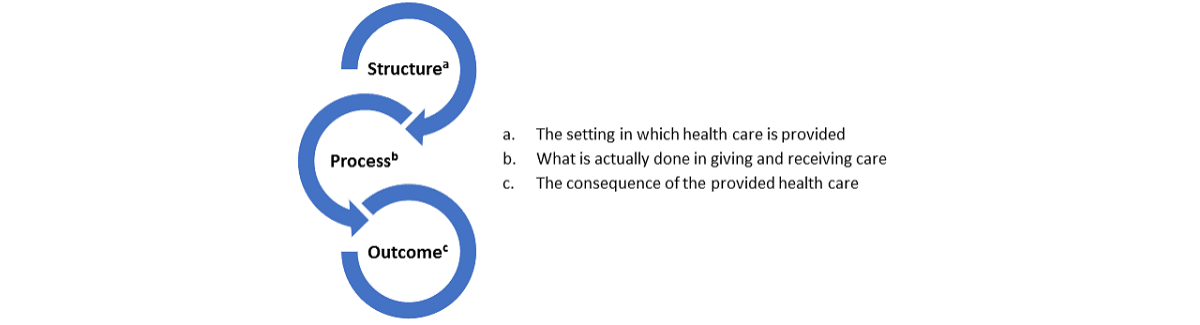
Donabedian Structure-Process-Outcome framework.

In the systematic literature review, the Donabedian Structure-Process-Outcome (SPO) framework was used to identify indicators related to the integration of eHealth into health care organizations [17,34-36] (Figure 1). According to Donabedian, health care quality is based on the aspects of these 3 categories and their relationships. The SPO framework and its categories are described in detail in a literature review [17].

In the literature review, we identified 111 potential indicators under the SPO categories that impact eHealth integration. The study demonstrated that 3 principles are important for successful integration. First, the patient’s role must be centrally placed in the organization of hybrid care. Second, technology must be well attuned to the organizational structure and daily care process. Third, the deployment of human resources must be aligned with desired results [17].

### Objectives

To translate the findings from the literature study into a modus operandi for health care organizations, we aimed to develop a model that can help health care organizations organize hybrid health care and identify improvement opportunities for a solid and sustainable integration of eHealth. To achieve this aim, the objectives of the concept mapping study included the following: (1) enrich and validate evidence-based knowledge from the literature review with practice-based knowledge from experts and (2) develop an SPO-based model for organizing hybrid health care with an accompanying self-assessment questionnaire.

## Methods

### Concept Mapping

Concept mapping is a highly structured methodology for organizing ideas from different stakeholders and other data sources to produce a common framework for complex topics that can be used for evaluation or planning [37-40]. The method integrates qualitative data collection with quantitative analysis to construct an interpretable pictorial view of different ideas and concepts and how these are interrelated [41,42]. Concept mapping has been used worldwide, for a diverse range of health care projects and studies to develop conceptual frameworks, as well as health and eHealth evaluations [43-49].

**Figure 2:**
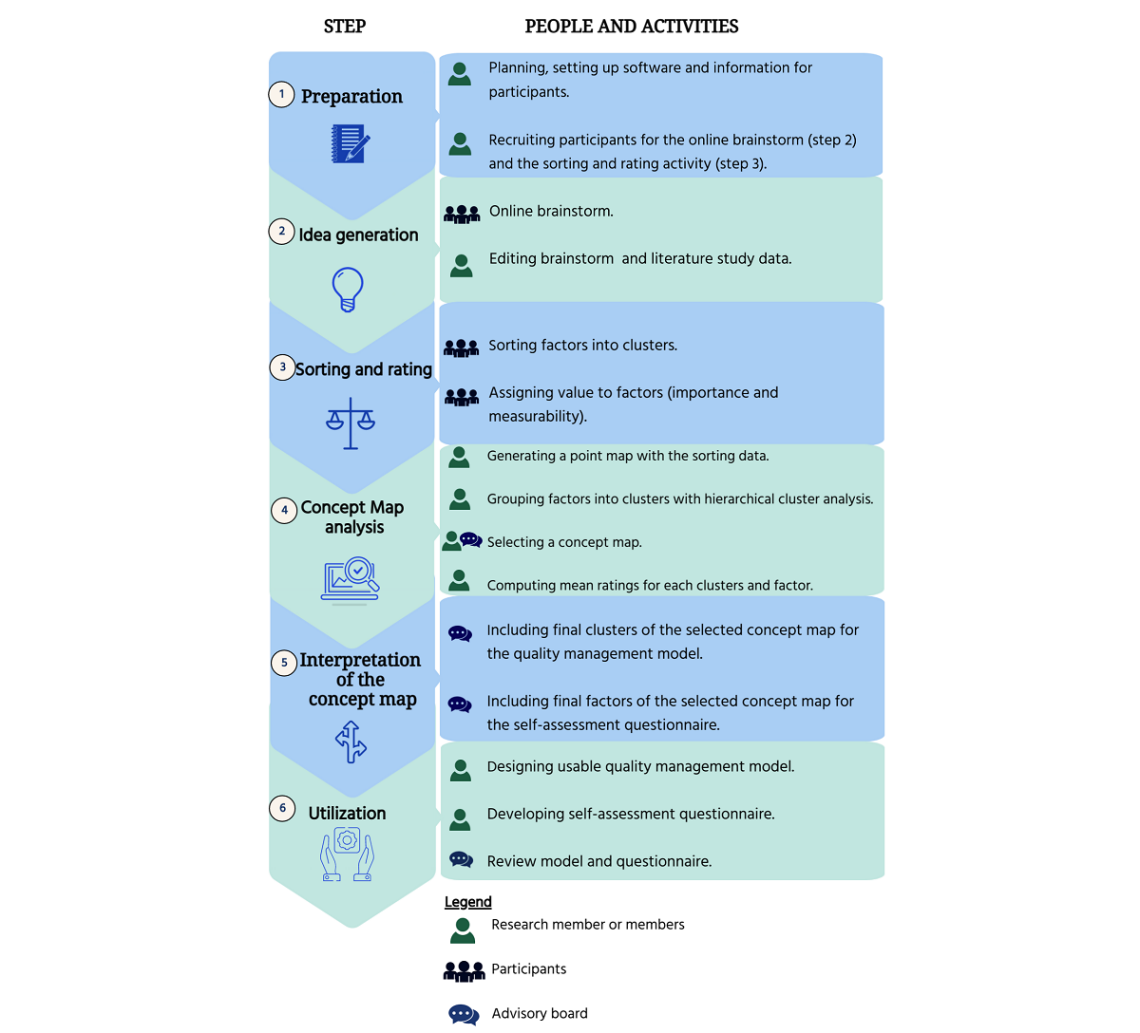
Concept mapping steps and study activities.

In this study, the 6-step concept mapping approach of Trochim and McLinden [42] was followed [49] to develop a usable, tailored, SPO-based quality management model for hybrid health care and an accompanying questionnaire. The six steps of concept mapping are as follows: (1) preparation, (2) idea generation, (3) sorting and rating, (4) concept mapping analysis, (5) map interpretation, and (6) utilization. Each step involves different activities leading to an output, which serves as an input for the next step. The steps and activities are explained in Figure 2 and in the paragraphs below. All the steps were supported by the GroupWisdom webtool [41,42].

### Step 1: Preparation

Concept mapping is most effective when multiple stakeholders participate in all the steps of the concept mapping process [50]*.* There is no strict limitation to the number of participants, ranging from small groups of 8 to 15 people to groups of hundreds of participants [50]. For this study, participants with eHealth experience, those employed by health care organizations, and patients with eHealth experience were recruited. The amount or kind of eHealth experience, health care setting, or disease was not relevant for inclusion. The goal was to create a diverse group in which different experiences, perceptions, and viewpoints complemented each other. We aimed to include a mix of health care professionals, patient experts (patients and caregivers), managers, directors, project leaders, researchers, and eHealth suppliers.

Potential participants were approached to attend both brainstorming in step 2 and sorting and rating in step 3. Participants were invited via the research team’s network, social media, and snowballing. Before agreeing to participate, participants received an information letter about the concept mapping method, the study’s purpose, and the SPO framework. None of the potential participants were familiar with our previous literature study results. A selected group was asked to participate in step 4 (concept mapping), step 5 (interpretation), and step 6 (utilization), which will be explained in the subsequent sections.

### Step 2: Idea Generation

#### Web-Based Brainstorming

In step 2, data from the participants were collected and combined with data from the literature study. Idea generation with participants was organized by brainstorming. Brainstorming is the most common method used in concept mapping, and can be either group brainstorming or individual brainstorming [42]. In this study, web-based brainstorming was conducted by the participants. Participants received a link via email with instructions, giving them access to the web-based brainstorm program of the GroupWisdom webtool. Before starting the brainstorming session, informed consent was provided, and participant characteristics (age, eHealth experience, professional background, and work setting) were collected to generate general background information about the participants. When the brainstorming started session, the following instruction was presented: “Name all factors, which you believe contribute to effective organization of patient care with eHealth, and what the outcomes of this care should be. Keep the ‘Structure-Process-Outcome’ framework in mind.”

For 23 days, the participants could list as many factors they considered essential contributors to effective hybrid health care. Participants could see each other’s inputs and save their brainstorming results in the meantime. They received reminders after 10 and 15 days.

#### Editing Brainstorming and Literature Study Data

After closing the web-based brainstorming session, the brainstorming and literature study data were combined for sorting and rating. A manageable amount of data for sorting and rating is ideally ≤100 to prevent redundancy and a loss of participants’ motivation [51,52]. To generate a final set of up to 100 factors, duplicates and factors that did not match the brainstorming instructions were removed. For this purpose, each factor was assessed independently by the authors, RT-S and ET-K. The assessments were compared, and disagreements were resolved by discussion between RT-S and ET-K. Next, RT-S edited the remaining factors for grammar and spelling.

Authors, MK and AR reviewed the editing process to check whether they would conclude the same selection and wording and made recommendations where appropriate. Finally, the set was entered into the GroupWisdom webtool, serving as an input for the sorting and rating activities.

### Step 3: Sorting and Rating

At the beginning of step 3, the participants received instructions for the sorting and rating tasks. For the sorting task, the participants were asked to cluster the factors into self-created clusters and assign names to the clusters. The participants were instructed to keep the Donabedian SPO categories in mind while sorting each factor into self-created clusters. For the rating task, each participant was asked to rate each factor by relevancy on a 5-point Likert scale, ranging from 1 (*not important at all* or *not feasible to measure*) to 5 (*very important* or *very feasible to measure*) by answering the questions, “How important is this factor for effective patient care with eHealth?” and “How feasible to measure is this factor?”

The participants had the opportunity to sort and rate over 3 weeks. They could save their activities and return later and received reminders after 10 and 15 days. The sorting data were approved for concept mapping analysis for participants who completed 75% of the sorting activity and created at least three clusters [41]. The rating data were included when the participant rated at least one factor.

### Step 4: Concept Mapping Analysis

Concept mapping analysis consisted of four main activities: (1) generating a point map with the sorting data, (2) grouping factors into clusters using hierarchical cluster analysis, (3) selecting a concept map from the hierarchical cluster analysis, and (4) computing average ratings for each factor and cluster of the selected concept map [50]. All computations were based on the concept mapping approach of Kane et al [53,54] and conducted using the GroupWisdom webtool.

#### Generating a Point Map With the Sorting Data

Data from the rating step were analyzed to create a point map [45,53,55,56]. A point map is a 2-dimensional point map, in which each point represents a factor [53]. The point map visually displayed the locations of all factors. Factors closer to each other on the point map were sorted together more frequently by the participants, whereas more distant factors on the map were sorted together less frequently [42,50,53]. The point map was constructed using a similarity matrix and multidimensional scaling algorithm. First, the similarity matrix indicated the number of times various factors were grouped together. Next, a multidimensional scaling algorithm plotted factors as points on a point map [42,54,55]. Subsequently, a stress value (0-1) was calculated, indicating the degree to which the distances on the point map fit the original similarity matrix [38,54]. The better the fit, the lower is the stress value.

#### Grouping Factors Into Clusters With Hierarchical Cluster Analysis

The point map provided the input for the hierarchical cluster analysis. The hierarchical cluster analysis grouped factors into clusters [44] using Ward algorithm [57]. The algorithm proposed several concept map solutions, where 2 clusters were merged at each following the proposed solution.

#### Selecting a Concept Map

From the proposed concept map solutions, a concept map that made sense for conceptualization was selected. There is no single correct number of clusters or mathematical decision criterion for selecting a concept map solution [38,56]. This study selected the number of clusters for the concept map by determining the range of the highest and lowest number of clusters. The range was the average number of clusters made by the participant and its SD.

Subsequently, the cluster solutions in this range were reviewed to select the cluster level by following the cluster tree in the Methods section of the studies by Trochim [53] and Kane et al [54]. Finally, in a meeting, 2 authors (RT-S and ET-K) and 2 participants reviewed the merging of clusters, beginning with the highest number of clusters and moving to the lowest. The 2 study participants were asked to join this meeting because of their extensive experience with eHealth, daily care processes, research, operational management, and concept mapping.

After establishing the number of clusters in the concept map, each factor was reviewed for compatibility with the cluster and to determine whether it was appropriate to move the factor to a different cluster. A cluster and its content were appropriate for inclusion when they were considered essential and usable for the quality management model [53].

In addition, each cluster received a name and description based on the cluster names that emerged from the sorting activity.

#### Computing Mean Ratings for Each Cluster and Factor of the Selected Concept Map

After the cluster map was selected, the relationships between ratings were computed using pattern-match and Go-zones [42].

Pattern-match and its Pearson product-moment (*r* value) were calculated to compare how the clusters of the selected concept map were rated on importance and measurability. The pattern-match visualized the mean ratings of each cluster in a ladder graph, connecting lines between the mean ratings on importance and measurable of each cluster [50,57]. The *r* value represented the correlation strength between the 2 mean ratings of all clusters [50,57].

Finally, multiple Go-zones were computed: a Go-zone of the total point map and Go-zones per cluster of the selected concept map. Go-zone is a 4-quadrant graph with an x-y graph [50], visualizing the mean ranking results of each factor on the questions “How important is this factor” and “How feasible to measure is this factor.” The minimum and maximum values for each axis were the minimum and maximum average Likert scores, respectively. The upper-right quadrant is called the G*o-zone* because it shows factors rated above the mean for both importance and measurability [42,58]. The pattern-match and Go-zone showed how important and measurable each cluster and its factors were rated for quality assessment by the individual participants during the step, sorting and rating.

The selected concept map, with its calculation of importance and measurability for each cluster and factor, formed the basis of interpretation in the next step [53].

### Step 5: Interpretation of the Concept Map

The selected concept map, with its pattern-match and Go-zones, was discussed with an advisory board. On the basis of the pattern-match and Go-zones, the advisory board decided which clusters and factors should be included in the quality management model and the accompanying questionnaire. The advisory board consisted of 4 study participants from the brainstorming and sorting step, of whom, 2 also participated in step 4, concept mapping analysis. The advisors were chosen because they could be future model users. In addition, all had extensive experience with eHealth, health care business, and as health care professionals (general practitioners, nurses, anesthetists, and clinical psychologists) in different health care settings.

The advisors voted individually on which clusters and factors of the selected concept map should be included in the quality management model and questionnaire to ensure usability. Using a web-based survey, the following questions were asked: “Which cluster should be included in the quality management model based on the mean cluster rating scores of the pattern matches? Please, specify your choice.” and “On which factors should the questionnaire give focus? Guide your choice by the Go-zones of each cluster and the Go-zone of the total point map. Please specify your choice.” The advisors could not see each other’s votes. By 75% (3/4) agreement or more, the concerned clusters and factors were operationalized in the quality assessment model and questionnaire. Where there was less agreement, the advisors viewed all responses, including the comments, and were asked to vote again. This process was repeated until a 75% consensus was reached. The web-based survey results were used as inputs to develop the quality management model and its questionnaire.

### Step 6: Utilization

#### Quality Management Model

The remaining clusters and their positions in the selected concept map provided the blueprint for the quality management model. First, the excluded clusters and factors were removed from the concept map. Second, the concept map with the remaining clusters was used to produce a logic model. A logic model is a framework that visualizes the interrelations between the clusters in graphic form and is therefore valuable for quality evaluation [59]. The SPO framework [34,35] was used to identify logical interrelationships between the clusters. Accordingly, noticeable SPO connections between the clusters were drawn on the map by RT-S. A simplified version of the logic model was designed for clarity and readability. Authors SW, ET-K, and RT-S discussed the design of the quality management model to ensure the usability and clarity of the model.

#### Self-assessment Questionnaire

The questionnaire was drafted by RT-S with the remaining factors, taking the advisors’ comments into account. The questionnaire should give care organizations insight into the quality of hybrid care and how quality develops over time. On the one hand, the questionnaire must be easy to use and uniformly independent of the type of health care organization, type of eHealth, and disease. On the other hand, the questionnaire results must provide specific guidance to improve the quality of specific clusters and factors.

The concept model and questionnaire were submitted to the advisors for peer review of usability and clarity. Their comments were processed by RT-S, resulting in an improved draft. Finally, ET-K and SW peer reviewed the last draft to ensure that the representatives’ comments were implemented entirely in the quality management model and the related questionnaire.

### Ethics Approval

Approval by an ethics committee was not needed because no intervention or trial has occurred in the sense that the research participants were subjected to actions or had modes of behavior imposed on them [60].

## Results

### Participant Characteristics (Step 1)

A total of 39 people participated in this study. The participants had a mean age of 45.2 (SD 11.1) years and were mainly working at the family medicine clinic (12/39, 31%) or hospital (10/39, 26%) within a management function (16/39, 41%) or as a health care professional (14/39, 36%). A total of 59% (23/39) of the participants estimated their eHealth experience to be extensive. The 3 most commonly used eHealth tools were apps (37/147, 25.2% participants), web portals (35/147, 23.8% participants), and video communication (34/147, 23.1% participants). An overview of the participants’ characteristics is shown in Table 1.

**Figure 3:**
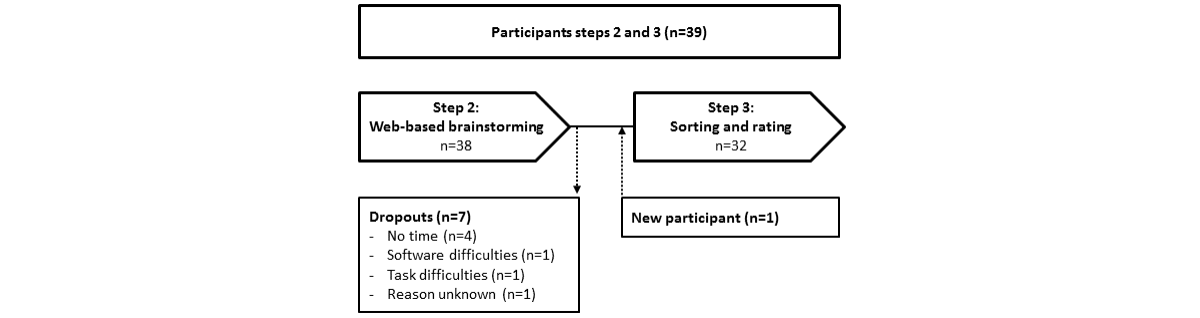
Number of participants at steps 2 and 3.

Of the 39 participants, 38 (97%) completed the brainstorming sessions. In all, 18% (7/38) of the participants dropped out after the brainstorming session, and a new participant joined the sorting and rating phase. In total, 79% (31/39) of the participants completed the sorting and rating phase (Figure 3).

**Table 1:** Participant characteristics (N=39).

Variables			Values
Age (years), mean (SD)			45.2 (11.1)
**Main work setting, n (%)**			
	Family medicine	12 (31)	
	Hospital	10 (26)	
	Mental health clinic	5 (13)	
	Nursing and residential care	5 (13)	
	eHealth supplier	4 (10)	
	Research institute	2 (5)	
	Patient experts (self-employed)	1 (3)	
**Main profession, n (%)^a^**			
	Manager, director, or project leader	16 (41)	
	Health care professional (eg, physician, nurse, therapist, or psychologist)	14 (36)	
	Patient expert (eg, patient or caregiver)	5 (13)	
	Researcher	3 (8)	
	Unknown	1 (3)	
**eHealth technology experience, n (%)^b^**			
	Apps	37 (25.2)	
	Web portals (eg, electronic health records or personal care records)	35 (23.8)	
	Video communication	34 (23.1)	
	Sensors and wearables	23 (15.6)	
	Artificial intelligence	13 (8.8)	
	Domotica and robotica	10 (6.8)	
**Estimated level of experience with eHealth, n (%)**			
	Extensive experience	23 (59)	
	Moderated experience	15 (38)	
	Limited experience	1 (3)	

^a^Many participants had dual roles, from which they were asked to choose one role.

^b^Participants could select multiple answers.

### Idea Generation (Step 2)

Brainstorming during idea generation resulted in a list of 203 factors. A total of 111 potential indicators were extracted from the literature study [17]. Both lists were aggregated, resulting in a list of 314 factors. Editing of the data led to a final list of 78 factors. These 78 factors served as inputs for the sorting and rating activity. The list of 78 factors is provided in Multimedia Appendix 1.

### Sorting and Rating (Step 3)

The rating data of the 32 participants were included in this study. All factors received mean rating scores of >3.1, for both importance and measurability. The mean ratings on the questions, “How important is this factor for successful integration of eHealth?” and “How feasible to measure is this factor” are described in Multimedia Appendix 1.

The sorting data of 8 people were excluded, with the reason “less than 75% sorted” (n=4, 50%) or “sorted in two clusters” (n=4, 50%). The mean number of clusters of the approved data was 7 (SD 3.5) with a range of 3 to 15 clusters.

### Concept Mapping Analysis (Step 4)

#### Visual Representation

**Figure 4:**
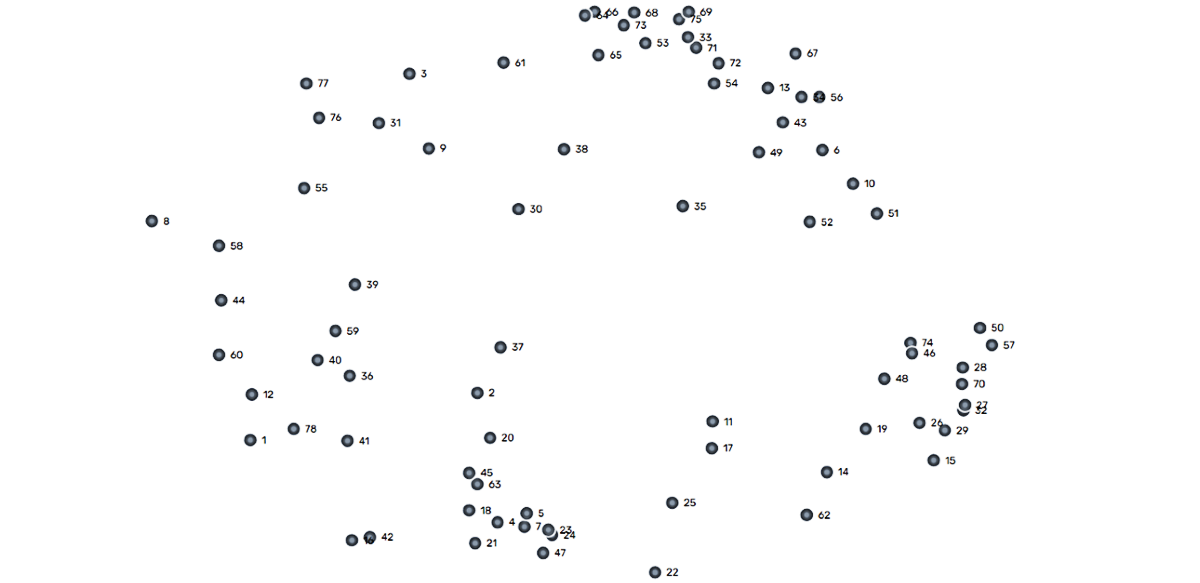
Point map.

The point map in Figure 4 shows how the 78 factors are related according to the sorting data. The point map had a stress value of 0.26, indicating that it had a good fit with the original similarity matrix [38,54].

The point map displays the locations of all factors that were frequently sorted closer together by the participants, whereas unrelated factors were plotted farther from each other. The number of points corresponds to the number of factors presented in Multimedia Appendix 1.

#### Selecting the Concept Map

**Figure 5:**
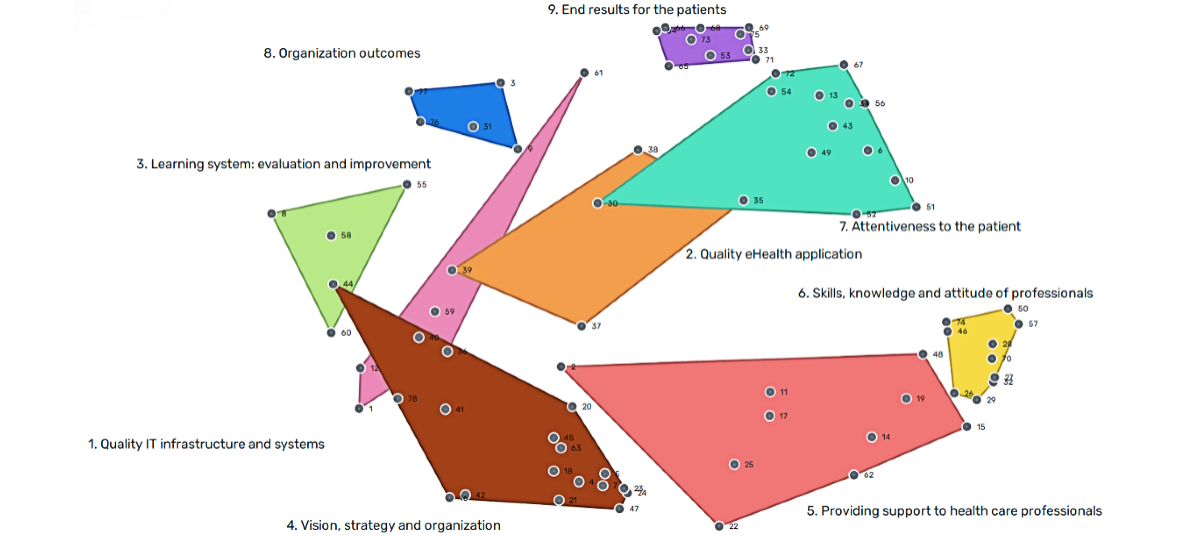
Nine-cluster concept map. IT: information technology.

Concept map solutions ranging from 11-cluster to 3-cluster options were reviewed (mean 7, SD 3.5). The 9-cluster concept map was selected to make the most sense of conceptualization. A few factors (n=14) were unanimously replaced, leading to the concept map shown in Figure 5. Replaced factors and their reasons are presented in Multimedia Appendix 2. The 9 clusters were labeled and received a short description, as described in Table 2. The number of points corresponds to the number of factors presented in Multimedia Appendix 1. The clusters represent how the participants sorted the factors into self-created clusters using the proposed cluster labels.

**Table 2:** Clusters labels and descriptions.

Cluster number^a^	Cluster label	Description	Included factors, n
1	Quality information technology infrastructure and systems	Conditions concerning technology, information technology systems, and data.	6
2	Quality eHealth application	Conditions concerning the eHealth application.	4
3	Learning system: evaluation and improvement	Evaluation and realignment with stakeholders and the patient care objectives for a continuous development.	4
4	Vision, strategy, and organization	Responsibilities of the health care organization concerning vision, strategy, policy, leadership, funding, and work process designs.	16
5	Providing support to health care professionals	Conditions arranged by the health care organization to encourage the use of eHealth among its health care professionals.	10
6	Skills, knowledge, and attitude of health care professionals	Health care professionals’ ability to provide hybrid care.	10
7	Attentiveness to the patient	Organize the daily care process in line with the patient’s needs, demand for care, and its capacity.	13
8	Organization outcomes	Outcomes for the health care organization; for example, quality health care provision and health care logistics.	5
9	End results for the patient	Outcomes for the patients; for example, health, added value, satisfaction, ownership, and convenience.	10

^a^Number corresponds with the number of the concerning cluster in Figure 5.

#### Mean Ratings for Each Cluster and Factor of the Selected Concept Map

**Figure 6:**
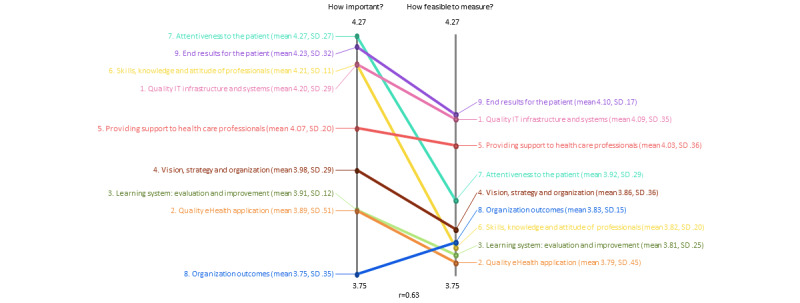
Pattern-match between the cluster-mean scoring on importance and measurability, with Pearson product-moment. IT: information technology.

The pattern-match showed that all clusters had a mean score between 3.75 and 4.27 on the importance and a mean score between 3.79 and 4.10 on measurability (Figure 6). The cluster with the highest mean score on importance was *Attentiveness to the patient* (mean 4.27, SD 0.27), and the cluster with the highest mean score on measurability was *End results for the patients* (mean 4.10, SD 0.17). On the contrary, the cluster with the lowest mean score on importance was *Organization outcomes* (mean 3.75, SD 0.36), whereas the cluster *Quality eHealth application* (mean 3.79, SD 0.45) had the lowest mean score on measurability. The *r* value was 0.63, indicating a predictable alignment between the rating of importance and the rating of measurability. The mean ratings of the factors and Go-zones per cluster are included in Multimedia Appendix 3.

### Interpretation of the Concept Map (Step 5)

The pattern-match and Go-zones were input to determine which clusters and factors of the selected concept map should be included in the quality management model and questionnaire. Decisions were made in 2 voting rounds. Of the 9 clusters, the cluster *Organization outcomes* was not included in the quality management model, based on the voting (3/4, 75%) of the advisors had doubts about including the cluster in the model) and after discussion with the research team. The factors included in the questionnaire concerned those placed in the Go-zone of the total point map or the Go-zone of the clusters. As a result, 8 clusters remained in the model and 33 factors in the questionnaire remained as a manageable utility for quality assessment (Textbox 1). Multimedia Appendix 3 presents the responses and comments of the advisory board during the voting rounds.

**The included clusters and factors.**

**Quality Information technology infrastructure and systems (1)**

- Information technology architecture available within the health care organization (1).
- Back-up scenario during technical problems (12).

**Quality eHealth application (2)**

- The eHealth application is user-friendly (35).

**Learning system: evaluation and improvement (3)**

- Cocreation: eHealth is developed, implemented and redeveloped with different stakeholders (8).
- Monitoring and evaluation of service and treatment results (58).

**Vision, strategy, and organization (4)**

- Support the implementation and development of eHealth in the organization with good project management (4).
- Mobilizing funding for working with eHealth (16).
- Clear internal policies regarding the use of eHealth (18).
- Vision supported by the line, “Why are we doing this?” (21).
- Care delivery with eHealth complies with laws and regulations (41).
- Financial reimbursements for eHealth deployment (42).
- Redesign the current work process and review what contributes to the desired care outcomes (47).

**Providing support toward health care professionals (5)**

- Health care professionals have easy access to information technology resources; for example, device, internet, screen, or headset (2).
- Embedding eHealth in the daily practice of health care professionals (11).
- Training and supervision for health care professionals (15).
- Help desk for health care professionals (17).
- Information on the treatment with eHealth is clear and accessible to the health care professional (19).

**Skills, knowledge, and attitude of health care professionals (6)**

- Good balance between face to face and eHealth for the health care professional (46).
- The health care professional has confidence in the eHealth application (70).
- The health care professional is satisfied with working with eHealth (74).

**Attentiveness to the patient (7)**

- Clear communication to the patient about how care is offered (10).
- Personalized care, considering patient needs with regard to (deployment of) eHealth (13).
- The patient has easy access to the necessary information technology resources; for example, device, Internet, and so on (30).
- Patients receive practical support in using the eHealth application; for example, a help desk (49).
- The patient has confidence in the eHealth application (67).
- The patient has the flexibility to use eHealth wherever and whenever it is convenient (72).

**End results for the patient (9)**

- The patient can integrate the use of eHealth in their daily life (33).
- Treatment with eHealth has a positive influence on the patient’s health (64).
- Treatment with eHealth contributes to the patient’s self-reliance (65).
- The patient is satisfied (68).
- The patient has easy access to care (71).
- eHealth provides logistical convenience for the patient (73).
- eHealth has added value for the patient (75).

### Utilization (Step 6)

#### Utilization Model

**Figure 7:**
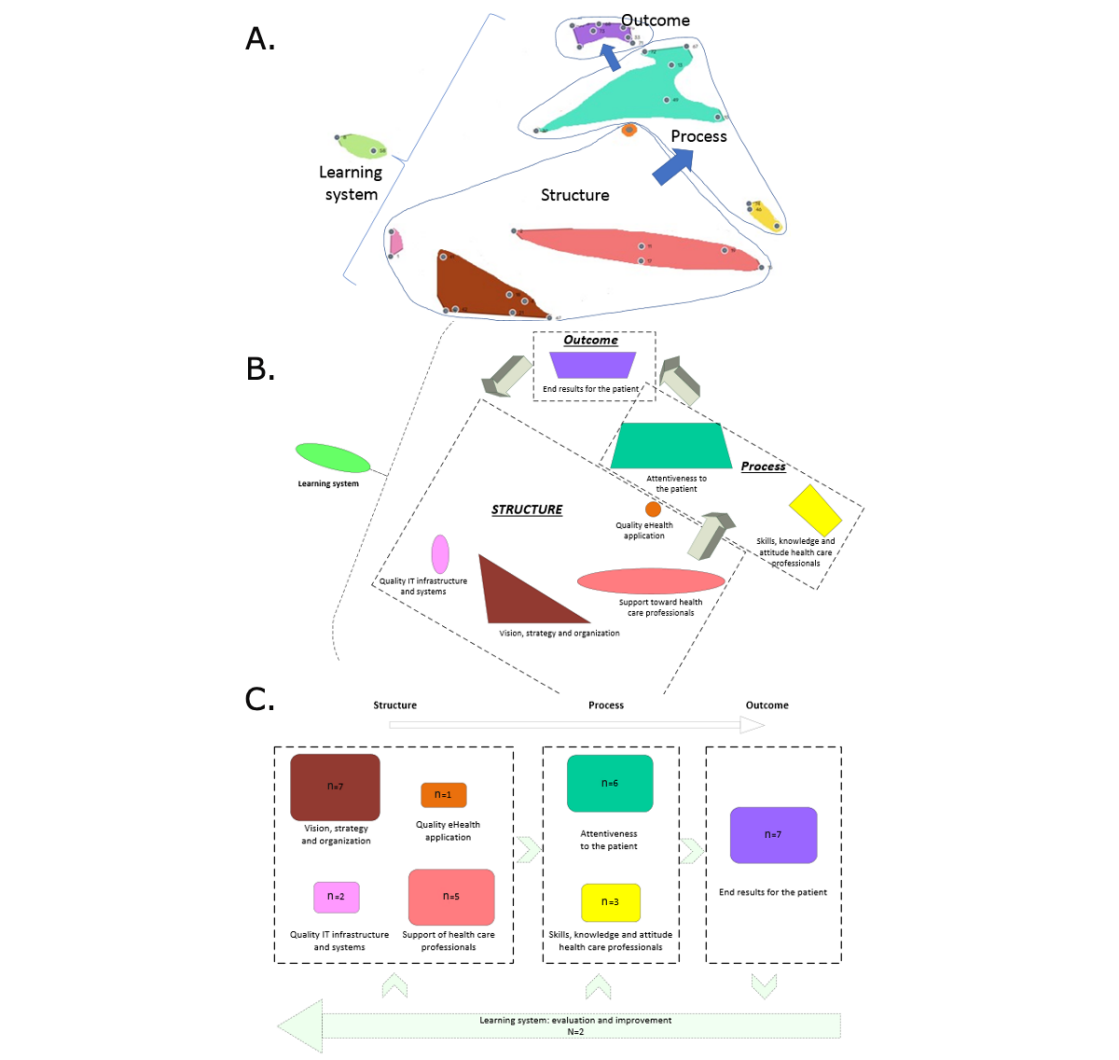
Simplification of the model. (A) Removing the excluded cluster and factors from the selected concept map and adding the overarching categories’ structure, process, and outcome. (B) Drawing a logic interrelationship with structure, process, and outcome categories. (C) Simplification into a quality management model. IT: information technology.

The clusters and factors excluded from the voting rounds were removed from the selected concept map. The remaining clusters (n=8) and their factors (n=33) led to nonoverlaying clusters on the concept map. Above the clusters, the SPO categories were positioned as overarching themes to emphasize the interrelations between the clusters. In addition, a complex cluster map can be simplified into a logic model. Figures 7A-C show the simplification of the model.

The overarching categories, *structure*, *process* and *outcomes* and the clusters’ interconnections refer to the Donabedian SPO framework [34,35]. The cluster *Learning system* is visualized in the arrows with the dashed line. The numbers inside the clusters represent the number of factors included.

#### Utilization Questionnaire

The remaining 33 factors were included in the questionnaire, where each factor can be measured on how effectively it is organized and developed over time. The advisory board noted that measuring the quality progress of hybrid health care is very important, in addition to learning and continuous improvement with stakeholders. Subsequently, the idea was to enrich the questionnaire with a quality progress tracker based on the plan-do-check-act (PDCA) cycles of Deming [61]. Incorporating the PDCA cycle makes it possible to assess the quality easily and uniformly with tailored feedback for health care organizations. PDCA is a well-known cycle method for continuous improvement and quality measurement [61]. The PDCA cycles assess each factor’s quality by measuring the extent to which *The objective is tangible?* (plan), *The plan is implemented?* (do), *To what extent is the plan realized?* (check), and *Providing feedback on the quality of the execution to make improvements* (act) [61]. Each factor can be monitored on the quality level of the PDCA cycles using a Likert score (0-10). A score of 0 means there is *no plan to improve the concerning factor*, and a score of 10 means *continue improvement with stakeholders*. The Likert scoring is based on the PDCA cycles and the 2 factors of the cluster Learning system, which include the following: (1) *Cocreation: eHealth is being developed and implemented with various stakeholders* and (2) *Monitoring and evaluation of service- and treatment outcomes*. Using the PDCA cycles in combination with a Likert score provides a health care organization insight into improvement possibilities for each factor or cluster.

Finally, the model and questionnaire obtained a more convenient workname Hybrid Health Care Quality Assessment (HHQA). The HHQA model and questionnaire with suggestions on how to use it are explained in Multimedia Appendix 4.

## Discussion

### Principal Findings

In this concept mapping study, we aimed to develop an SPO-based model and an accompanying self-assessment questionnaire for hybrid health care. By combining practice-based knowledge from eHealth users with an evidence-based literature review, we found that organizational, technological, and process and personal factors affect the quality of hybrid health care. Health care organizations must understand that these factors play a role in organizing hybrid health care and should be familiar with ways to improve them. The authors developed the HHQA, which can be used to systematically assess and improve the quality of hybrid health care.

The HHQA model includes 8 clusters. Cluster 1 (*Vision, strategy, and organization*) includes the responsibilities of the management to set the vision, strategy, policy, leadership, finance, and project management. Cluster 2 (*Quality information technology infrastructure and systems*) focuses on information technology infrastructure and back-up scenarios by information technology issues. Cluster 3 (*Quality eHealth application*) concerns the user-friendliness of the digital health application itself. Cluster 4 (*Providing support toward care professional*) and cluster 5 (*Skills, knowledge, and attitude of health care professionals*) include factors concerning health care providers. Cluster 4 focuses on factors that should be arranged for the individual health care professional by the care organization, and cluster 5 includes the responsibilities of the professional. The patient is central in cluster 6 (*Attentiveness to the patient*). This cluster contains the measurement of factors that allow patients to increase their self-management and consider the individual patient’s needs. Patient centeredness is also reflected in cluster 7 (*Patient outcomes*), including factors such as patient’s health outcomes, added value, satisfaction, ownership, and convenience. Finally, cluster 8 (*Learning system*), forms the relationship between the continued development of hybrid health care with stakeholders and health care provision objectives. The factors in cluster 8 provide insight into where alignment can be improved with other organizational criteria and actions, such as cost-benefit or capacity management.

The interdependencies of the clusters are logically expressed in the HHQA model because of the overarching categories of the Donabedian SPO framework. Moreover, according to eHealth users, clusters consist of the most important factors for the quality of hybrid health care. Using the questionnaire, each factor (33 in total) was measured to determine how effectively it was organized and developed over time. Subsequently, the main results of the questionnaire were shown at the cluster level. It was possible to zoom in on the relevant factors for each cluster.

### Comparison With Literature

In our previous literature review [17], we concluded that the capabilities of patients, health care professionals, and technology play a crucial role in the quality of hybrid health care. We also concluded that offering hybrid health care requires adjusting the daily care process and appropriate process monitoring. The conclusions from the literature review are reflected in the HHQA clusters, namely, the patient’s role is visible in the clusters *Attentiveness to the patient* and *Patient outcomes*; the health care professional’s role is central in the clusters *Providing support toward health care professionals* and *Skills, knowledge, and attitude of professionals*; and technology is covered in the clusters *Quality information technology infrastructure and systems* and *Quality eHealth application*. The adjustment of the daily care processes is elaborated in the cluster *Vision, strategy, and organization.* Finally, monitoring is embedded in the cluster *Learning system* and the PDCA-progress tracker.

The 8 clusters of the HHQA model fit the 3 overarching categories of the Donabedian SPO framework. According to Donabedian [34], health care quality is based on aspects of these 3 categories and their relationships. The interaction between the categories can be bidirectional and is an “unbroken chain of antecedents, followed by intermediate ends, which are themselves the means to still further ends” [35]. Our research translated the complex interaction between the categories, structure, process, and outcome into user language.

The HHQA connects essential contributions to the quality of hybrid health care using a progress tracker. The relationship between quality contributors and continuous improvement also appears in the European Foundation for Quality Management Model (EFQM) [62,63]; nonadoption, abandonment, scale-up, spread, sustainability (NASSS) [32]; and the Consolidated Framework for Implementation Research (CFIR) [64,65]. All models approach the organizational structure, process, and outcomes with continuous improvement in a structured manner, but with different focus areas. For example, the EFQM is not specified for health care, in contrast to the NASSS and CFIR. The NASSS focuses on the adoption of technology and reduces implementation complexity, whereas the CFIR emphasizes on implementation in general. However, none of them have been specified for quality assessment and improvement of hybrid health care.

Nevertheless, it is interesting to conduct a detailed examination of the assessment questionnaires of the EFQM and NASSS. The EFQM deployed the Results-Approach-Deployed-Assessment-Refinement (RADAR) method [66,67], a questionnaire to assess the quality improvement at each EFQM criteria, which incorporates the continued improvement circle. The assessment using the RADAR method is similar to the PDCA cycle in our questionnaire, as both monitor continuous quality improvement by completing the cycle plan-executing-monitoring and refining. However, the RADAR, similar to the EFQM model, is not specified for hybrid health care. In addition, the NASSS comes with a questionnaire to monitor the complexity of technology implementation in health care [68], but the focus is on project management instead of the hybrid health care process itself. Furthermore, there are other questionnaires measuring the quality of eHealth [69-72] or the quality of health care [73,74]. However, these questionnaires are concerned with the quality assessment of eHealth nationwide [68,70], the quality of a specific digital health application [70,72], or measuring the quality of a specific disease pathway [73,74]. To the best of our knowledge, HHQA is the first questionnaire measuring the quality of hybrid health care at an organizational level, taking the role of the patient, health care professionals, and technology into account, accompanied by an improvement progress tracker. Therefore, the authors recommend using the HHQA to measure and improve the quality of hybrid health care.

### Strengths and Limitations

This study has several strengths. First, the HHQA was developed in cocreation with stakeholders who are direct users of eHealth. Therefore, the HHQA content was drawn from inside the health care system itself and not conceived or imposed outside the health care organizations. Second, stakeholders choose the included clusters and factors. The researcher only played a facilitating role. Consequently, the clusters and factors accurately reflect stakeholders’ views and values, expressed in their own words and visual representations. Third, the stakeholder group was diverse and consisted of representatives of health care professionals, patients, managers, researchers, and eHealth designers. Nevertheless, the stress value of the point map shows that the stakeholders’ outcomes are highly compatible. Therefore, the study results are likely to be generalizable to everyday practices. Fourth, the model and questionnaire were developed by combining scientific and practice-based knowledge. Together, these strengths result in important factors for effective hybrid health care covering different users' needs and organization requirements.

Our study had some limitations. First, the questionnaire had not yet been tested in health care organizations. This will be conducted in a follow-up study. Although eHealth users from different health care organizations have reviewed the model and questionnaire, the model and questionnaire may still be too abstract for daily practice, as is often the case in scientific research [75-77]. A follow-up study could provide concrete recommendations on how to use the HHQA. Second, it is conceivable that other factors and clusters could be included in other participants and health care environments. We attempted to overcome this problem by creating diverse groups of participants with different backgrounds, various eHealth experiences, and different kinds of health care settings. In addition, combining idea generation through brainstorming with results from a systematic literature review reduces the risk of bias. Third, based on the analysis of the concept mapping phase, 14 factors were moved to other clusters. However, some of these factors were moved far across the map, which was not entirely in line with the spirit of group concept mapping. Nevertheless, we deemed it necessary to move these factors for substantive reasons. Fourth, the advisory group consisted of 4 participants. We wanted to avoid overquestioning the participants and, therefore, deliberately selected a group of delegates who reflected on the diversity among the participants and who also had experience with quality management and concept mapping. Combined with in-depth preparation and discussion among the research groups, this appeared to be the most feasible solution.

Finally, it is worth pointing out that the HHQA gives a first general impression of improvement, as there is much to be gained in taking the role of the patient, health care professionals, and used technology into account [17]. Furthermore, the authors will continue with follow-up research and warm-heartedly welcome repetition of the study to improve the HHQA, taking into account the different users and health care environments.

### Conclusions

This study developed a quality management model and an accompanying self-assessment questionnaire tailored for hybrid health care, the HHQA. A quality model for hybrid care is indispensable for effectively integrating eHealth into regular care and delivering high-quality health care. The HHQA covers all relevant aspects for the assessment and sustainable improvement of hybrid health care and the interrelations of eHealth with organizational, technical, and human factors. The next step is to validate and apply the HHQA model and questionnaire in practice.

## Appendix

Multimedia Appendix 1 Mean (SD) rating scores of clusters and factors.

Multimedia Appendix 2 Relocation factors and their reasons.

Multimedia Appendix 3 Results voting “which clusters and factors to include” and given comments.

Multimedia Appendix 4 Suggestion utilization Hybrid Health Care Quality Assessment questionnaire.

## Abbreviations

CFIR: Consolidated Framework for Implementation Research
EFQM: European Foundation for Quality Management Model
HHQA: Hybrid Health Care Quality Assessment
NASSS: nonadoption, abandonment, scale-up, spread, sustainability
PDCA: plan-do-check-act
RADAR: Results-Approach-Deployed-Assessment-Refinement
SPO: Structure-Process-Outcome
