# Supplementary material for: Development of a Quality Management Model and Self-assessment Questionnaire for Hybrid Health Care: Concept Mapping Study
Source: JMIR Form Res. 2022 Jul 7;6(7):e38683. doi: 10.2196/38683 (PMC9305399; doi:10.2196/38683)
Supplement: Multimedia Appendix 1 [file formative_v6i7e38683_app1.docx]

**APPENDIX 1: MEAN (SD) RATING OF EACH CLUSTER AND FACTOR**

**Table 1.** Mean (SD)ᵃ rating of the clusters and factors

| Cluster | Nr | Factor | How important?ᵇ  Mean (SD) | How feasible to measure?ᶜ  Mean (SD) |
| --- | --- | --- | --- | --- |
| 1. Quality information technology infrastructure and systems | | | 4.20(0.29) | 4.09 (0.35) |
|  | 12 | Back -up scenario during technical problems. | 4.21 (0.59) | 4.17 (0.99) |
|  | 1 | IT architecture available within the health care organization. | 4.13 (0.73) | 4.36 (0.93) |
|  | 36 | Technology is up-to date and works flawlessly. | 4.56 (0.57) | 3.88 (0.97) |
|  | 59 | Use of reliable data. | 4.42 (0.64) | 3.74 (0.99) |
|  | 40 | Exchange of data possible between different systems; for example, EPD, HIS, HIC. | 4.25 (0.88) | 3.73 (1.14) |
|  | 61 | Built-in patient notifications. | 3.64 (0.64) | 4.67 (0.75) |
| 2. Quality eHealth application | | | 3.89 (0.51) | 3.79 (0.2) |
|  | 35 | The eHealth application is user-friendly. | 4.75 (0.43) | 4.38 (0.70) |
|  | 39 | The eHealth application is suitable as a medical intervention. | 3.39 (1.15) | 3.96 (0.89) |
|  | 37 | The provision of care with eHealth is evidence-based. | 3.73 (0.86) | 3.71 (1.10) |
|  | 38 | The eHealth application is also usable when the care needs are different than expected. | 3.68 (0.87) | 3.13 (1.05) |
| 3. Learning system: evaluation and continue improvement of hybrid care | | | 3.91 (0.12) | 3.81 (0.25) |
|  | 8 | Co-creation: eHealth is developed, implemented and redeveloped with different stakeholders. | 4.09 (0.72) | 3.91 (1.00) |
|  | 58 | Monitoring and evaluation of service and treatment results. | 3.91 (0.67) | 4.17 (0.75) |
|  | 60 | Learn from each other through 'best and worst practices' or other forms of exchanging experiences. | 3.75 (0.80) | 3.58 (1.22) |
|  | 55 | Use data to tailor the treatment to the patient's situation. | 3.88 (0.84) | 3.58 (1.04) |
| 4. Vision, strategy, and organization | | | 3.98 (0.29) | 3.86 (0.36) |
|  | 41 | Care delivery with eHealth complies with laws and regulations. | 4.33 (0.69) | 4.67 (0.47) |
|  | 16 | Mobilizing funding for working with eHealth. | 4.33 (0.90) | 3.96 (1.16) |
|  | 21 | Vision supported by the line, “Why are we doing this?” | 4.33 (0.62) | 3.86 (0.87) |
|  | 4 | Support the implementation and development of eHealth in the organization with good project management. | 4.29 (0.84) | 4.00 (1.00) |
|  | 47 | Redesign the current work process and review what contributes to the desired care outcomes. | 4.23 (0.52) | 4.00 (0.80) |
|  | 18 | Clear internal policies regarding the use of eHealth. | 4.17 (0.80) | 3.96 (1.00) |
|  | 42 | Financial reimbursements for eHealth deployment. | 4.09 (0.85) | 3.95 (1.07) |
|  | 23 | Leadership: share the vision, mission and strategy to create support. | 4.08 (0.93) | 3.79 (1.00) |
|  | 7 | Set eHealth goals in your organization. | 3.96 (0.72) | 4.42 (0.70) |
|  | 5 | Achievement of organizational goals concerning eHealth is invested in the organization. | 3.95 (0.93) | 3.96 (0.86) |
|  | 78 | The costs of treatment with eHealth are transparent. | 3.46 (0.87) | 4.00 (0.88) |
|  | 24 | Create urgency and direction within the organization: make eHealth part of every innovation and health care project. | 3.96 (0.86) | 3.57 (1.10) |
|  | 20 | eHealth has added value for the strategy of the organization. | 3.78 (0.93) | 3.71 (1.14) |
|  | 63 | Organize the work process in such a way that it becomes almost impossible to make mistakes. | 3.73 (0.91) | 3.22 (1.06) |
|  | 45 | There is good collaboration with external partners. | 3.54 (0.76) | 3.26 (0.94) |
|  | 44 | Treatment with eHealth is in line with community and regional needs and developments. | 3.52 (0.76) | 3.46 (1.15) |
| 5. Providing support to health care professionals | | | 4.07 (0.19) | 4.03 (0.36) |
|  | 15 | Training and supervision for health care professionals. | 4.25 (0.60) | 4.46 (0.82) |
|  | 2 | Health care professionals have easy access to IT resources; for example, device, internet, screen, headset. | 4.25 (0.59) | 4.17 (0.85) |
|  | 17 | Helpdesk for health care professionals. | 4.24 (0.64) | 4.71 (0.54) |
|  | 19 | Information on the treatment with eHealth is clear and accessible to the health care professional. | 4.13 (0.60) | 4.09 (0.65) |
|  | 11 | Embedding eHealth in the daily practice of health care professionals. | 4.29 (0.73) | 4.00 (0.87) |
|  | 14 | Staff are given time to (learn to) work with eHealth. | 4.17 (0.69) | 3.91 (0.79) |
|  | 62 | Clear guidelines and protocols for health care professionals. | 3.88 (0.59) | 4.20 (0.80) |
|  | 48 | Make the work easier for the health care professional. | 3.92 (0.86) | 3.74 (0.94) |
|  | 25 | Strong collaboration concerning eHealth with your colleagues of different departments within your health care organization. | 3.91 (0.72) | 3.50 (0.78) |
|  | 22 | Encourage and support the use of eHealth by 'ambassadors' in the teams. | 3.70 (0.86) | 3.54 (1.29) |
| 6. Skills, knowledge, and attitude of professionals | | | 4.21 (0.11) | 3.82 (0.20) |
|  | 70 | The health care professional has confidence in the eHealth application. | 4.40 (0.63) | 4.13 (0.80) |
|  | 46 | Good balance between face-to-face and eHealth for the health care professional. | 4.29 (0.68) | 4.13 (1.01) |
|  | 74 | The health care professional is satisfied with working with eHealth. | 4.12 (0.65) | 4.04 (0.81) |
|  | 29 | The health care professional is willing to learn to work with eHealth. | 4.32 (0.67) | 3.72 (1.04) |
|  | 28 | The health care professional recognizes the added value of eHealth. | 4.33 (0.62) | 3.68 (0.93) |
|  | 27 | The health care professional feels comfortable working with eHealth. | 4.17 (0.83) | 3.83 (0.85) |
|  | 50 | Health care professionals focus on supporting patients' self-management in their treatment. | 4.20 (0.75) | 3.73 (1.09) |
|  | 26 | Health care professionals are digitally literate. | 4.13 (0.53) | 3.78 (1.02) |
|  | 57 | The health care professional can adapt to the changing relationship and needs of the patient. | 4.09 (0.78) | 3.55 (1.23) |
|  | 32 | The health care professional knows at which moments in the care process, the patient can be supported with eHealth. | 4.04 (0.54) | 3.64 (0.98) |
| 7. Attentiveness to the patient | | | 4.27 (0.27) | 3.92 (0.29) |
|  | 13 | Personalized care: taking into account patient needs regard to (deployment of) eHealth. | 4.58 (0.49) | 3.96 (0.91) |
|  | 10 | Clear communication to the patient about how care is offered. | 4.50 (0.50) | 4.33 (0.69) |
|  | 67 | The patient has confidence in the eHealth application. | 4.48 (0.64) | 4.12 (0.71) |
|  | 43 | Care with eHealth meets the needs of the target population. | 4.36 (0.56) | 3.74 (0.67) |
|  | 49 | Patients receive practical support in using the eHealth application; for example, a help desk. | 4.21 (0.41) | 4.54 (0.64) |
|  | 72 | The patient has flexibility to use eHealth where and when it is convenient. | 4.16 (0.83) | 4.09 (1.02) |
|  | 30 | The patient has easy access to the necessary IT resources; for example, device, internet. | 4.16 (0.77) | 3.95 (1.02) |
|  | 6 | Attention to patient eHealth literacy. | 4.17 (0.70) | 3.55 (1.08) |
|  | 56 | The patient is open to treatment with eHealth. | 4.43 (0.71) | 3.83 (0.82) |
|  | 52 | Clear expectations between patient and practitioner. | 4.40 (0.63) | 3.68 (1.09) |
|  | 54 | There is personal attention for the patient. | 4.38 (0.70) | 3.91 (0.88) |
|  | 51 | Prior to treatment, an assessment is made of whether eHealth can work for this patient. | 4.22 (0.66) | 3.55 (1.12) |
|  | 34 | The patient is supported by his or her environment in the use of eHealth. | 3.48 (0.85) | 3.67 (0.99) |
| 8. Organization outcomes | | | 3.75 (0.35) | 3.83 (0.15) |
|  | 3 | eHealth leads to increased quality of health care services. | 4.17 (0.55) | 3.96 (0.89) |
|  | 76 | Improvement of health care logistics; for example, waiting time, turnaround time, drop out, no-show, time per treatment. | 3.88 (0.88) | 4.04 (0.95) |
|  | 31 | eHealth contributes to more meaningful care. | 4.00 (0.75) | 3.73 (1.09) |
|  | 9 | eHealth provides opportunities to offer care in a more enjoyable way. | 3.50 (0.65) | 3.65 (1.09) |
|  | 77 | eHealth affects the referrals rate. | 3.20 (0.75) | 3.78 (0.98) |
| 9. End results for the patient | | | 4.23 (0.32) | 4.10 (0.17) |
|  | 75 | eHealth has added value for the patient. | 4.76 (0.43) | 4.18 (0.83) |
|  | 68 | The patient is satisfied. | 4.42 (0.64) | 4.40(0.63) |
|  | 65 | Treatment with eHealth contributes to the patient's self-reliance. | 4.28 (0.70) | 4.17 (0.85) |
|  | 66 | Improved patient quality of life. | 4.39 (0.64) | 3.92 (0.91) |
|  | 33 | The patient can integrate the use of eHealth in his or her daily life. | 4.32 (0.68) | 4.09 (0.79) |
|  | 64 | Treatment with eHealth has a positive influence on the patient's health. | 4.32 (0.68) | 4.04 (0.81) |
|  | 73 | eHealth provides logistical convenience for the patient. | 4.22 (0.66) | 4.25 (0.78) |
|  | 71 | The patient has easy access to care. | 4.18 (0.57) | 4.17 (0.75) |
|  | 69 | The patient is satisfied with the knowledge and skills of the health care professional. | 3.95 (0.64) | 4.00 (0.72) |
|  | 53 | Patient is therapy compliant. | 3.48 (0.96) | 3.75 (0.94) |

ᵃ The numbers are rounded to two decimal places.

ᵇ “How important is this factor for effective patient care with eHealth?”

ᶜ “How feasible to measure is this factor?”
