# Supplementary material for: Development of a Quality Management Model and Self-assessment Questionnaire for Hybrid Health Care: Concept Mapping Study
Source: JMIR Form Res. 2022 Jul 7;6(7):e38683. doi: 10.2196/38683 (PMC9305399; doi:10.2196/38683)
Supplement: Multimedia Appendix 2 [file formative_v6i7e38683_app2.docx]

**APPENDIX 2. RELOCATION FACTORS AND ITS REASON**

**Table 1.** Relocation factors and descriptions of the reasons

| **Factor** | | **Original cluster** | | **Transfer to cluster** | | **Reason transfer** |
| --- | --- | --- | --- | --- | --- | --- |
| 26. | Health care professionals are digitally literate. | 5. | Providing support to health care professionals | 6. | Skills, knowledge, and attitude of professionals | Digital literacy is a skill. |
| 29. | The health care professional is willing to learn to work with eHealth. | 5. | Providing support to health care professionals | 6. | Skills, knowledge, and attitude of professionals | Willing to learn is an attitude. |
| 30. | The patient has easy access to the necessary IT resources; for example, device, internet. | 2. | Quality eHealth application | 7. | Attentiveness to the patient | Easy access to IT-resources concerns not the eHealth application itself but is a condition for access to eHealth. |
| 39. | The eHealth application is suitable as a medical intervention. | 1. | Quality IT infrastructure and systems | 2. | Quality eHealth application | It concerns the eHealth application itself. |
| 41. | Care delivery with eHealth complies with laws and regulations. | 1. | Quality IT infrastructure and systems | 4. | Vision, strategy, and organization | It concerns the vision, strategy, and how to organise hybrid health care. |
| 44. | Treatment with eHealth is in line with community and regional needs and developments. | 3. | Learning system: evaluation and continue improvement of hybrid care | 4. | Vision, strategy, and organization | It concerns the vision, strategy, and how to organise hybrid health care. |
| 48. | Make the work easier for the health care professional. | 6. | Skills, knowledge, and attitude of professionals | 5. | Providing support to health care professionals | It concerns a condition that a health care organization has to arrange. |
| 54. | There is personal attention for the patient. | 9. | End results for the patient | 7. | Attentiveness to the patient | Personal attention is needed during the treatment. |
| 55. | Use data to tailor the treatment to the patient's situation. | 8. | Organization outcomes | 3. | Learning system: evaluation and continue improvement of hybrid care | Tailoring the treatment to the patients’ situation is needed during the treatment. |
| 60. | Learn from each other through 'best and worst practices' or other forms of exchanging experiences | 1. | Quality IT infrastructure and systems | 3. | Learning system: evaluation and continue improvement of hybrid care | It concerns learning and improving. |
| 61. | Built-in patient notifications. | 8. | Organization outcomes | 1. | Quality IT infrastructure and systems | Notifications has to be built in the IT-systems. |
| 72. | The patient has flexibility to use eHealth where and when it is convenient. | 9. | End results for the patient | 7. | Attentiveness to the patient | Using eHealth is needed during the treatment. |
| 78. | The costs of treatment with eHealth are transparent. | 1. | Quality IT infrastructure and systems | 4. | Vision, strategy, and organization | Providing transparent costs of hybrid health care is a part of the vision, strategy, and organization. |
