# Supplementary material for: Development of a Quality Management Model and Self-assessment Questionnaire for Hybrid Health Care: Concept Mapping Study
Source: JMIR Form Res. 2022 Jul 7;6(7):e38683. doi: 10.2196/38683 (PMC9305399; doi:10.2196/38683)
Supplement: Multimedia Appendix 3 [file formative_v6i7e38683_app3.docx]

**APPENDIX 3: RESULTS OF THE VOTINGS ‘WHICH CLUSTERS AND FACTORS TO INCLUDE IN THE MODEL AND QUESTIONNAIRE’AND GIVEN COMMENTS BY THE ADVISORY BOARD**

This appendix consists of two parts. The first part concerns the inclusion of the clusters in the quality management model. The second part concerns the inclusion of the factors in the accompanying questionnaire.

## Results voting on which clusters the selected concept map should be included in the quality management model

**Table 1.** Results ‘Which clusters should be included in the model?’

| **Which Clusters** | | **First round** | | | **Second Round** |
| --- | --- | --- | --- | --- | --- |
|  | | **Yes (%)** | **No (%)** | **I don’t know (%)** | **Yes**ᶜ **(%)** |
| 1 | Quality IT infrastructure and systems | 75 | 25 | 0 |  |
| 2 | Quality eHealth application | 75 | 25 | 0 |  |
| 3 | Learning system: evaluation and improvement | 50 | 0 | 50 | 75 |
| 4 | Vision, strategy, and organization | 100 | 0 | 0 |  |
| 5 | Providing support to health care professionals | 100 | 0 | 0 |  |
| 6 | Skills, knowledge and attitude health care professionals | 75 | 25 | 0 |  |
| 7 | Attentiveness to the patient | 100 | 0 | 0 |  |
| 8 | Organization outcomes | 0 | 25 | 75 |  |
| 9 | End results for the patient | 75 | 25 | 0 |  |

ᵃ By more than 75% agreement, the cluster is operationalized in the quality management model resp. questionnaire.

ᵇ Cluster nr 3; ‘learning system’ and factors at questions nr. 2, 6-8 were asked in the second round.

ᶜ Percentage based on four people, including one no response.

**Comments advisory group (the comments were placed unanimously) per cluster**

Cluster 1. Quality IT infrastructure and systems

*“You cannot deliver eHealth without a good quality system.”*

*“It is challenging to integrate hybrid care into care processes without thinking carefully about your infrastructure.”*

*“This can also become a prerequisite that needs to be established once. There is so much investment in IT that this is probably already high and not prohibitive.”*

Cluster 2. Quality eHealth application

*“Because it determines the outcome for the patient.”*

*“Quality and eHealth applications are transient. Therefore, it should be considered per model, but not in a guideline.“*

*“An obvious limiting factor for implementation is that it is quickly done if it does not work (well).”*

Cluster 3. Learning system: evaluation and improvement

Ronde 1:

*“Evaluation is of importance but possible as a second step.”*

*“Because we are in transition, it is precisely learning and a learning attitude essential for getting better. It is never right the first time.”*

Ronde 2:

*“I believe measurability is important because it can help organizations demonstrate the effects of digitization within the health care.”*

Cluster 4. Vision, strategy, and organization

*“Embedding from within the organization is necessary.”*

*“A vision and strategy to support the workflow are essential for proper embedding in an organization.”*

*“With a vision, strategy and organization, the Board of directors focuses on hybrid care and thus directs the organization. It is then no longer a toy.”*

Cluster 5. Providing support to care professionals

*“To develop a positive attitude among health care professionals, support in applications is necessary.”*

*“Without proper guidance, health care professionals will drop out at the slightest setback.”*

Cluster 6**.** Skills, knowledge, and attitude care professionals

*“It can be merged with cluster 5.”*

*“Not only are the buttons of the application needed but also learning to work with a patient remotely and support them in the use of e-health.”*

Cluster 7. Attentiveness to the patient

*“Hybrid care should take into account the patient's wishes and abilities.”*

*“Personalize care also in e-health because every patient differs in economic circumstances -equipment- education and ability to do things. Eye for the difference.”*

Cluster 8. Organization outcomes

*“Relevant, but possibly just less relevant than the other parameters.”*

*“For me, cluster 9 is less important.”*

*“There is a much less straight line between e-health application and organization outcomes, at most some proxies like fewer repeat consultations.”*

Cluster 9. End results for the patient

*“Departure and added value to the patient, both measurable and important.“*

*“Bit analogous to cluster 7.”*

*“The patient must always be taken into account. Both in the ability and inability.“*

*This is the most important outcome of all: if there is no result here, it can go in the garbage can.*

## Results voting on which factors of the selected concept map should be included in the questionnaire

**Table 2.** Results ‘Which factors should be included in the model?’

| **Which factors** | | **First roundᵃ** | **Second Round** |
| --- | --- | --- | --- |
|  | | **Yes (%)** | **Yes**ᶜ **(%)** |
| 1 | All factors | 0 |  |
| 2ᵇ | All factors in the Go-zone of each *cluster* or the Go-zone of the *total point* map with 78 factors. | 50 | 50ᶜ |
| 3 | All factors in the zone ‘*importance*’ of *each cluster* and in the *zone ‘importance’ of* the *total point map* with 78 factors, regardless the measurability. | 0 |  |
| 4 | All factors in the Go-zone*’* of the *total point map* with 78 factors. | 0 |  |
| 5 | All factors in the zone ‘i*mportance*’ of the *total point map* with the 78 factors, whatever its measurability | 0 |  |
| 6ᵇ | All factors in the Go-zone of *each cluster* | 25 | 0ᶜ |
| 7ᵇ | All factors in the zone ‘*importance*’ of *each* *cluster*, whatever its measurability | 25 | 0ᶜ |
| 8ᵇ | Other, namely ....  Could be option 2, 6 or 7. It depends on the way it is incorporated into the questionnaire. | 0 | 25ᶜ |

ᵃ By more than 75% agreement, the factors are operationalized in the quality management model resp. questionnaire.

ᵇ Cluster nr 3; ‘learning system’ and factors at questions nr. 2, 6-8 were asked in the second round.

ᶜ Percentage based on four people, including one no response.

**Explanation visualization Go-zones at the tables of each cluster (next pages)**

Below each cluster tables stands the concerning Go-zone. The x-axis of the graph represents the mean ratings of the factors on the question “How important is this factor to successful patient care with eHealth” (in Dutch). The y-axis represents the mean rating of the factors on the question “How feasible to measure is this factor”? The upper-right quadrant (color green) is called the ‘Go-zone’ because it showed factors that were rated above the mean in both importance and measurability of the concerning cluster. In the lower-left quartile are the factors that have been scored as less important and measurable (color blue). The corresponding color of the zone is reflected in the tables. On the left side of each table, the color of the factor corresponds to the Go-zone of the belonging clusters.

In addition to the Go-zone per cluster, all factors are placed in a Go-zone with all 78 factors together. In this Go-zone, some factors just fall into a different quartile. The Go-zone with 78 factors is too large and cluttered to show it. Instead, the color of the corresponding quartile is shown on the right side of the table with the title "Quadrant 78 factors’.

The included factors are situated in the Go-zone of the concerning clusters (green in the left column of the table) or the Go-zone of the total point map (green in the right column of the table).

#### Cluster 1.  Quality information technology infrastructure and systems

| **Description** | | **Rating scores** | | **Quadrant**  **78 factors** |
| --- | --- | --- | --- | --- |
| Conditions concerning technology, IT systems and data. | | Importance | Measurability |  |
| **Nr.** | **Factors** | **Mean (SD)** | **Mean (SD)** |  |
| 12 | Back -up scenario during technical problems. | 4.21 (0.6) | 4.17 (1.0) |  |
| 36 | Technology is up-to date and works flawlessly | 4.56 (0.6) | 3.88 (1.0) |  |
| 59 | Use of reliable data. | 4.42 (0.6) | 3.74 (1.0) |  |
| 40 | Exchange of data possible between different system; for example, EPD, HIS, HIC. | 4.25 (0.9) | 3.73 (1.1) |  |
| 1 | IT architecture available within the health care organization | 4.13 (0.7) | 4.36 (0.9) |  |
| 61 | Built-in patient notifications. | 3.64 (0.6) | 4.67 (0.8) |  |


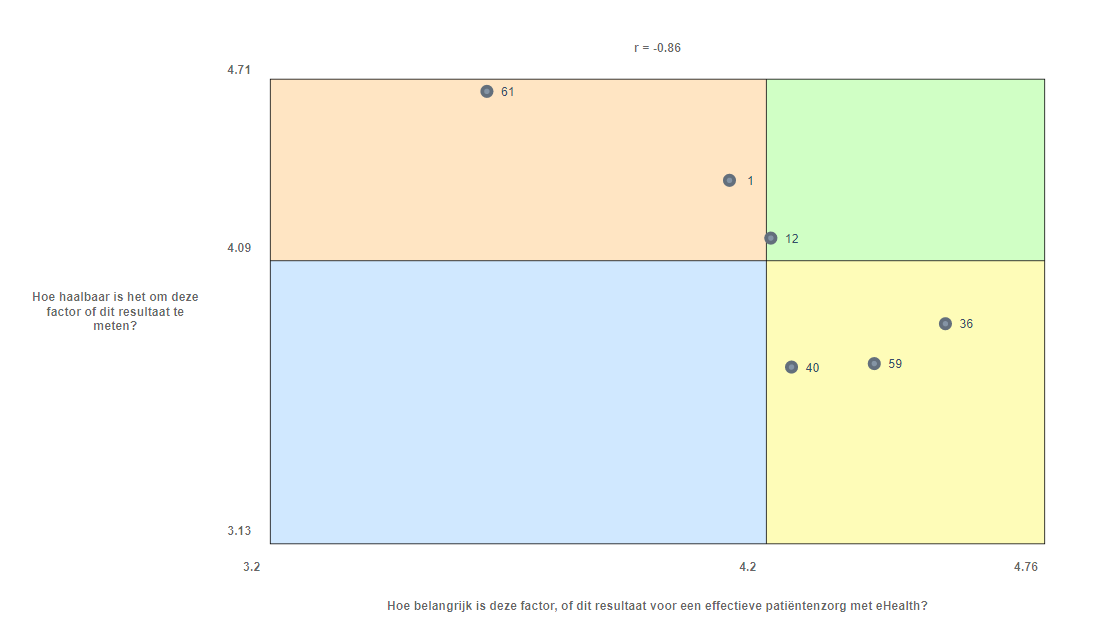


**Figure 1**. Go-zone cluster 1. Quality IT infrastructure and systems.

#### Cluster 2. Quality eHealth application

| **Description** | | **Rating scores** | | **Quadrant 78 factors** |
| --- | --- | --- | --- | --- |
| Conditions concerning the eHealth application. | | Importance | Measurability |  |
| **Nr.** | **Factors** | **Mean (SD)** | **Mean (SD)** |  |
| 35 | The eHealth application is user-friendly. | 4.75 (0.4) | 4.38 (0.7) |  |
| 39 | The eHealth application is suitable as a medical intervention. | 3.39 (1.2) | 3.96 (0.9) |  |
| 37 | The provision of care with eHealth is evidence-based. | 3.73 (0.9) | 3.71 (1.1) |  |
| 38 | The eHealth application is also usable when the care needs are different than expected. | 3.68 (0.9) | 3.13 (1.1) |  |


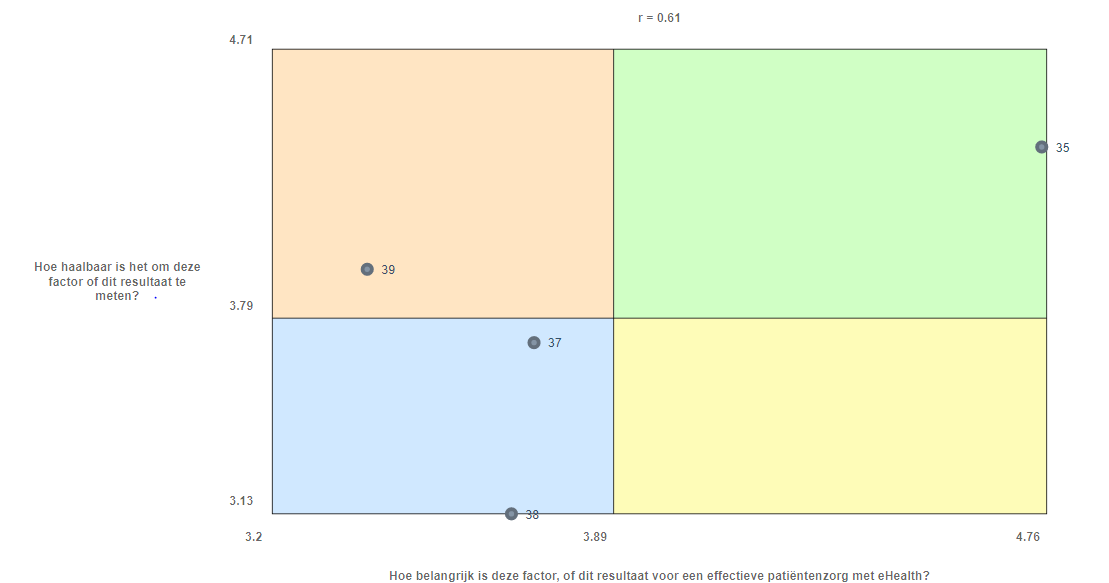


**Figure 2**. Go-zone cluster 2. Quality eHealth application.

#### Cluster 3. Learning system: evaluation and improvement

| **Description** | | **Rating scores** | | **Quadrant 78 factors** |
| --- | --- | --- | --- | --- |
| Evaluate and re-align with stakeholders and the patient care objectives for an continue development. | | Importance | Measurability |  |
| **Nr.** | **Factors** | **Mean (SD)** | **Mean (SD)** |  |
| 8 | Co-creation: eHealth is developed implemented and redeveloped with different stakeholders. | 4.09 (0.7) | 3.91 (1) |  |
| 58 | Monitoring and evaluation of service and treatment results. | 3.91 (0.7) | 4.17 (0.8) |  |
| 60 | Learn from each other through 'best and worst practices' or other forms of exchanging experiences. | 3.75 (0.8) | 3.58 (1.2) |  |
| 55 | Use data to tailor the treatment to the patient's situation. | 3.88 (0.8) | 3.58 (1.0) |  |


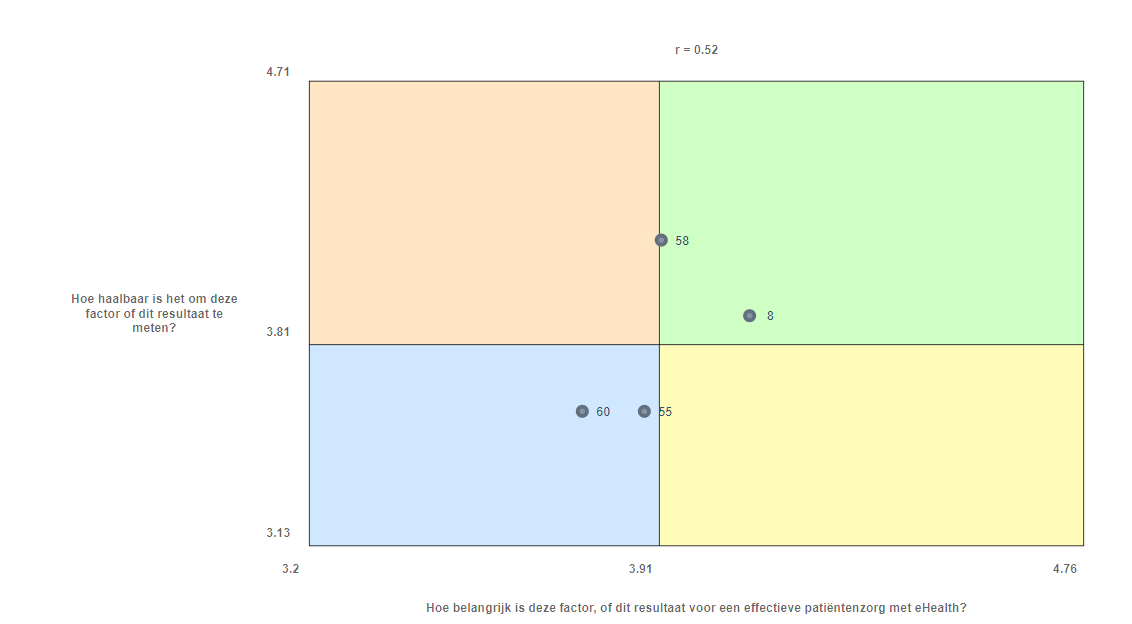


**Figure 3**. Go-zone cluster 3. Learning system: evaluation and improvement.

#### Cluster 4. Vision, strategy, and organization

| **Description** | | **Rating scores** | | **Quadrant 78 factors** |
| --- | --- | --- | --- | --- |
| Responsibilities of the health care organization concerning vision, strategy, policy, leadership, funding, and work process designs. | | Importance | Measurability |  |
| **Nr.** | **Factors** | **Mean (SD)** | **Mean (SD)** |  |
| 41 | Care delivery with eHealth complies with laws and regulations. | 4.33 (0.7) | 4.67 (0.5) |  |
| 16 | Mobilizing funding for working with eHealth. | 4.33 (0.9) | 3.96 (1.2) |  |
| 21 | Vision supported by the line, “Why are we doing this?” | 4.33 (0.6) | 3.86 (0.9) |  |
| 4 | Support the implementation and development of eHealth in the organization with good project management. | 4.29 (0.8) | 4.00 (1.0) |  |
| 47 | Redesign the current work process and review what contributes to the desired care outcomes. | 4.23 (0.5) | 4.00 (0.8) |  |
| 18 | Clear internal policies regarding the use of eHealth. | 4.17 (0.8) | 3.96 (1) |  |
| 42 | Financial reimbursements for eHealth deployment. | 4.09 (0.9) | 3.95 (1.1) |  |
| 23 | Leadership: share the vision, mission, and strategy to create support. | 4.08 (0.9) | 3.79 (1.0) |  |
| 7 | Set eHealth goals in your organization. | 3.96 (0.7) | 4.42 (0.7) |  |
| 5 | Achievement of organizational goals concerning eHealth is invested in the organization. | 3.95 (0.9) | 3.96 (0.9) |  |
| 78 | The costs of treatment with eHealth are transparent. | 3.46 (0.9) | 4.00 (0.9) |  |
| 24 | Create urgency and direction within the organization: make eHealth part of every innovation and health care project. | 3.96 (0.9) | 3.57 (1.1) |  |
| 20 | eHealth has added value for the strategy of the organization. | 3.78 (0.9) | 3.71 (1.1) |  |
| 63 | Organize the work process in such a way that it becomes almost impossible to make mistakes. | 3.73 (0.9) | 3.22 (1.1) |  |
| 45 | There is good collaboration with external partners. | 3.54 (0.8) | 3.26 (0.9) |  |
| 44 | Treatment with eHealth is in line with community and regional needs and developments. | 3.52 (0.8) | 3.46 (1.2) |  |


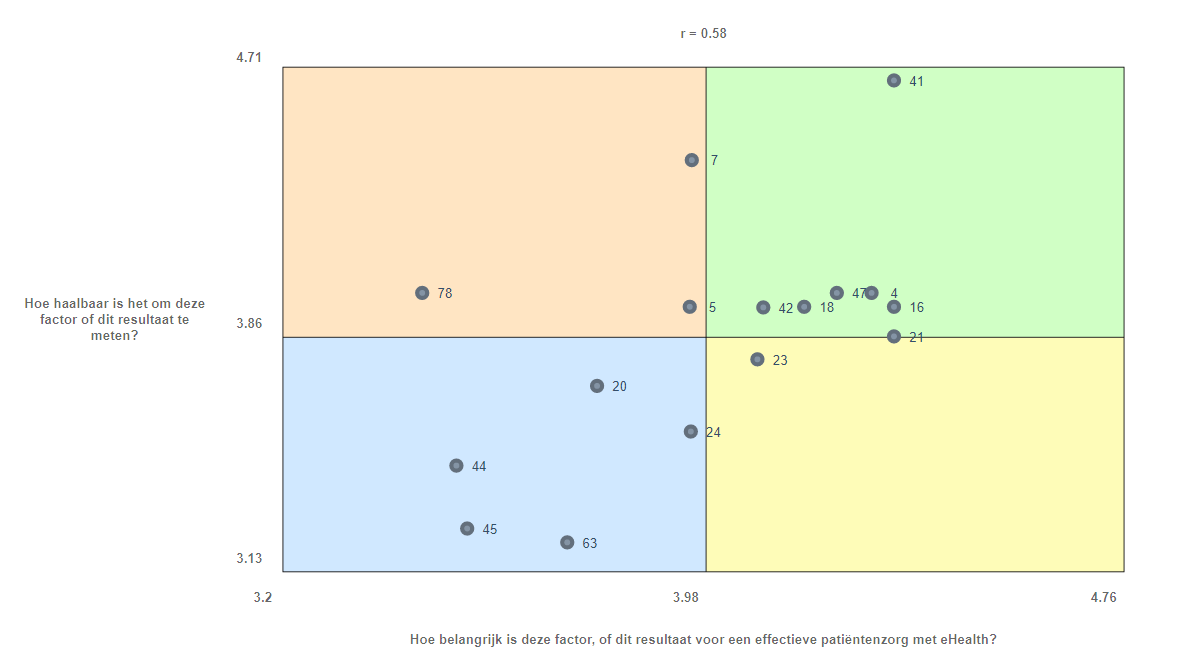


**Figure 4**. Go-zone cluster 4. Vision, strategy, and organization.

#### Cluster 5. Providing support to health care professionals

| **Description** | | **Rating scores** | | **Quadrant 78 factors** |
| --- | --- | --- | --- | --- |
| Conditions arranged by the health care organization to encourage the use of eHealth among its health care professionals. | | Importance | Measurability |  |
| **Nr.** | **Factors** | **Mean (SD)** | **Mean (SD)** |  |
| 15 | Training and supervision for health care professionals. | 4.25 (0.6) | 4.46 (0.8) |  |
| 2 | Health care professionals have easy access to IT resources; for example, device, internet, screen, headset | 4.25 (0.6) | 4.17 (0.9) |  |
| 17 | Helpdesk for health care professionals. | 4.24 (0.6) | 4.71 (0.5) |  |
| 19 | Information on the treatment with eHealth is clear and accessible to the health care professional. | 4.13 (0.6) | 4.09 (0.7) |  |
| 11 | Embedding eHealth in the daily practice of health care professionals. | 4.29 (0.7) | 4.00 (0.9) |  |
| 14 | Staff are given time to (learn to) work with eHealth. | 4.17 (0.7) | 3.91 (0.8) |  |
| 62 | Clear guidelines and protocols for health care professionals. | 3.88 (0.6) | 4.20 (0.8) |  |
| 48 | Make the work easier for the health care professional. | 3.92 (0.9) | 3.74 (0.9) |  |
| 25 | Strong collaboration concerning eHealth with your colleagues of different departments within your health care organization. | 3.91 (0.7) | 3.50 (0.8) |  |
| 22 | Encourage and support the use of eHealth by 'ambassadors' in the teams. | 3.70 (0.9) | 3.54 (1.3) |  |


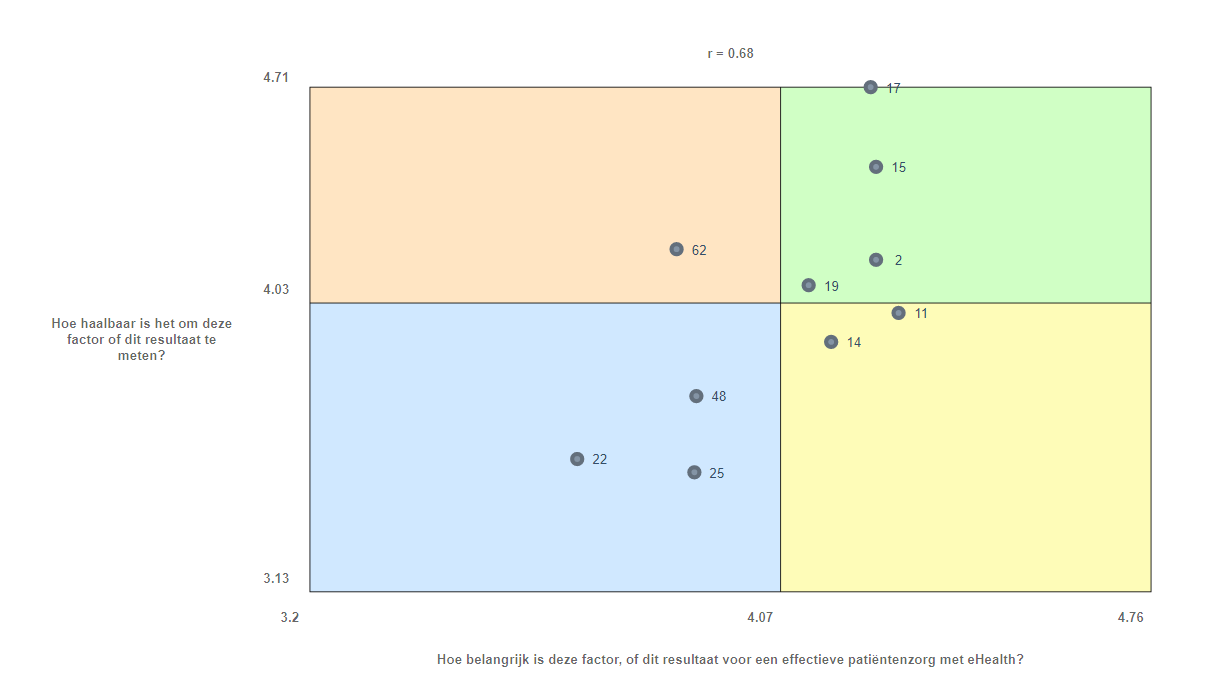


**Figure 5**. Go-zone cluster 5. Providing support toward health care professionals.

#### Cluster 6. Skills, knowledge, and attitude of professionals

| **Description** | | **Rating scores** | | **Quadrant 78 factors** |
| --- | --- | --- | --- | --- |
| Health care professional's ability to provide hybrid care. | | Importance | Measurability |  |
| **Nr.** | **Factors** | **Mean (SD)** | **Mean (SD)** |  |
| 70 | The health care professional has confidence in the eHealth application. | 4.40 (0.6) | 4.13 (0.8) |  |
| 46 | Good balance between face-to-face and eHealth for the health care professional. | 4.29 (0.7) | 4.13 (1.0) |  |
| 29 | The health care professional is willing to learn to work with eHealth. | 4.32 (0.7) | 3.72 (1.0) |  |
| 28 | The health care professional recognizes the added value of eHealth. | 4.33 (0.6) | 3.68 (0.9) |  |
| 27 | The health care professional feels comfortable working with eHealth. | 4.17 (0.8) | 3.83 (0.9) |  |
| 74 | The health care professional is satisfied with working with eHealth. | 4.12 (0.7) | 4.04 (0.8) |  |
| 50 | Health care professionals focus on supporting patients' self-management in their treatment. | 4.20 (0.8) | 3.73 (1.1) |  |
| 26 | Health care professionals are digitally literate. | 4.13 (0.5) | 3.78 (1.0) |  |
| 57 | The health care professional can adapt to the changing relationship and needs of the patient. | 4.09 (0.8) | 3.55 (1.2) |  |
| 32 | The health care professional knows at which moments in the care process, the patient can be supported with eHealth. | 4.04 (0.5) | 3.64 (1.0) |  |


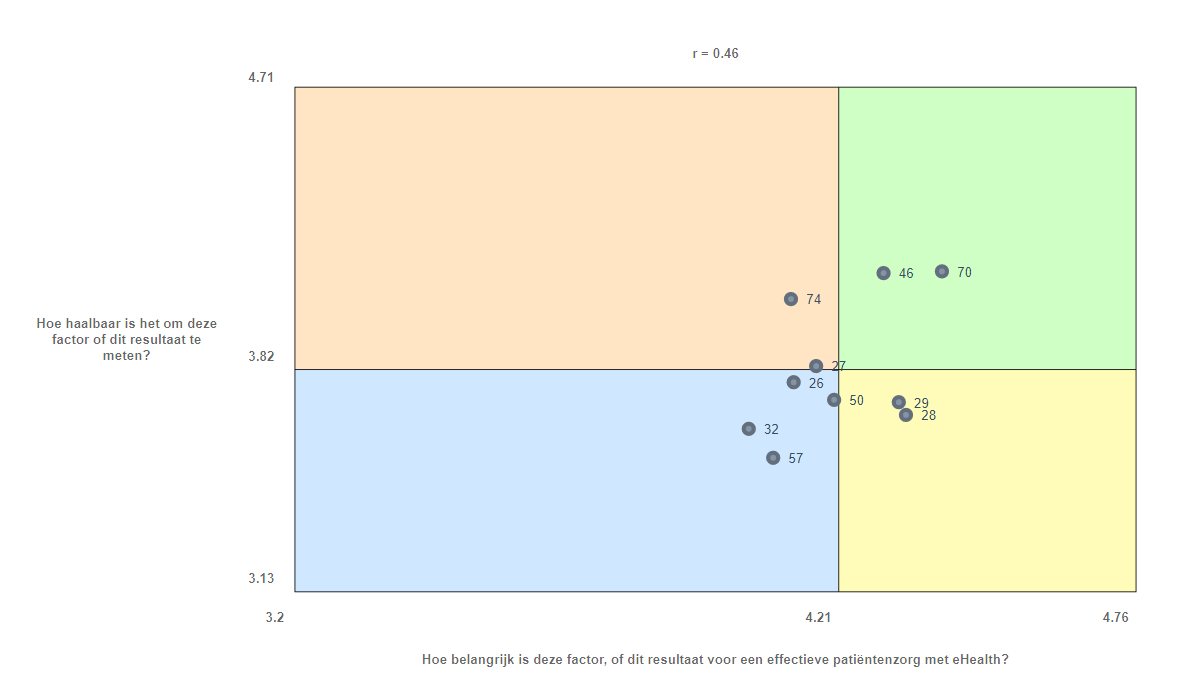


**Figure 6.** Go-zone cluster 6. Skills, knowledge, and attitude of professionals.

#### Cluster 7. Attentiveness to the patient

| **Description** | | **Rating scores** | | **Quadrant 78 factors** |
| --- | --- | --- | --- | --- |
| Organize the daily care process in line with the patient's needs, demand for care, and its capacity. | | Importance | Measurability |  |
| **Nr.** | **Factors** | **Mean (SD)** | **Mean (SD)** |  |
| 6 | Attention to patient eHealth literacy. | 4.17 (0.7) | 3.55 (1.1) |  |
| 13 | Personalized care, considering patient needs with regard to (deployment of) eHealth | 4.58 (0.5) | 3.96 (0.9) |  |
| 10 | Clear communication to the patient about how care is offered. | 4.50 (0.5) | 4.33 (0.7) |  |
| 67 | The patient has confidence in the eHealth application. | 4.48 (0.6) | 4.12 (0.7) |  |
| 56 | The patient is open to treatment with eHealth. | 4.43 (0.7) | 3.83 (0.8) |  |
| 52 | Clear expectations between patient and practitioner. | 4.40 (0.63) | 3.68 (1.1) |  |
| 54 | There is personal attention for the patient. | 4.38 (0.7) | 3.91 (0.9) |  |
| 43 | Care with eHealth meets the needs of the target population. | 4.36 (0.6) | 3.74 (0.7) |  |
| 49 | Patients receive practical support in using the eHealth application; for example, a help desk. | 4.21 (0.4) | 4.54 (0.6) |  |
| 72 | The patient has flexibility to use eHealth where and when it is convenient. | 4.16 (0.83) | 4.09 (1.02) |  |
| 30 | The patient has easy access to the necessary IT resources; for example, device, internet. | 4.16 (0.8) | 3.95 (1.0) |  |
| 51 | Prior to treatment, an assessment is made of whether eHealth can work for this patient. | 4.22 (0.7) | 3.55 (1.12) |  |
| 34 | The patient is supported by his or her environment in the use of eHealth. | 3.48 (0.9) | 3.67 (1.0) |  |


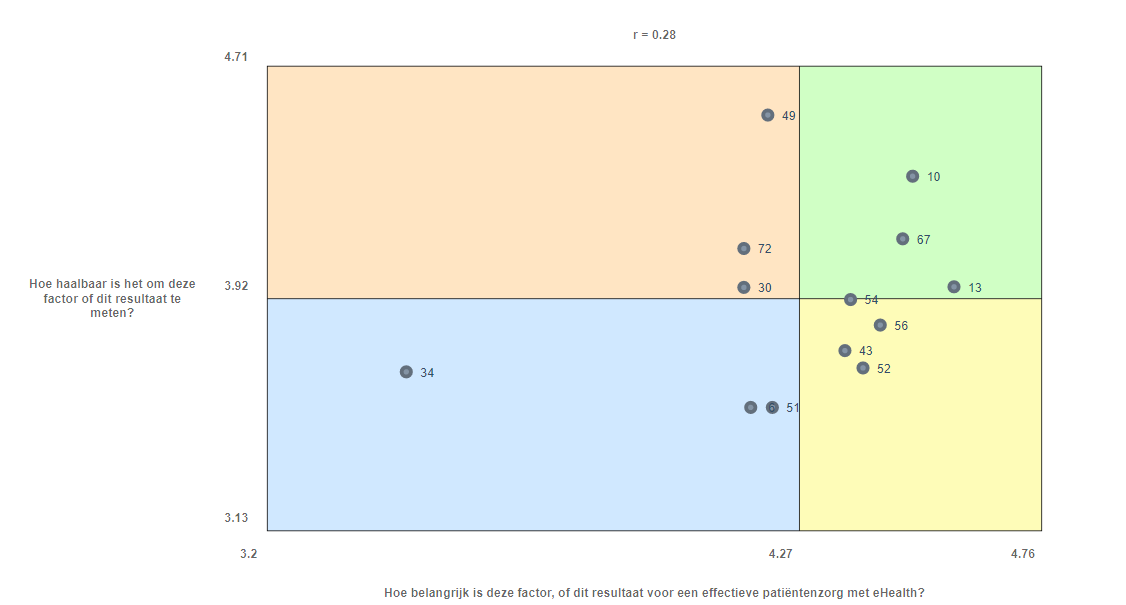


**Figure 7**. Go-zone cluster 7. Attentiveness to the patient.

#### Cluster 8. Organization outcomes

| **Description** | | **Rating scores** | | **Quadrant 78 factors** |
| --- | --- | --- | --- | --- |
| Outcomes for the health care organization. | | Importance | Measurability |  |
| **Nr.** | **Factors** | **Mean (SD)** | **Mean (SD)** |  |
| 3 | eHealth leads to increased quality of health care services. | 4.17 (0.6) | 3.96 (0.9) |  |
| 76 | Improvement of health care logistics; for example, waiting time, turnaround time, drop out, no-show, time per treatment. | 3.88 (0.9) | 4.04 (1.0) |  |
| 31 | eHealth contributes to more meaningful care. | 4.00 (0.8) | 3.73 (1.1) |  |
| 9 | eHealth provides opportunities to offer care in a more enjoyable way. | 3.50 (0.7) | 3.65 (1.1) |  |
| 77 | eHealth affects the referrals rate. | 3.20 (0.8) | 3.78 (1.0) |  |


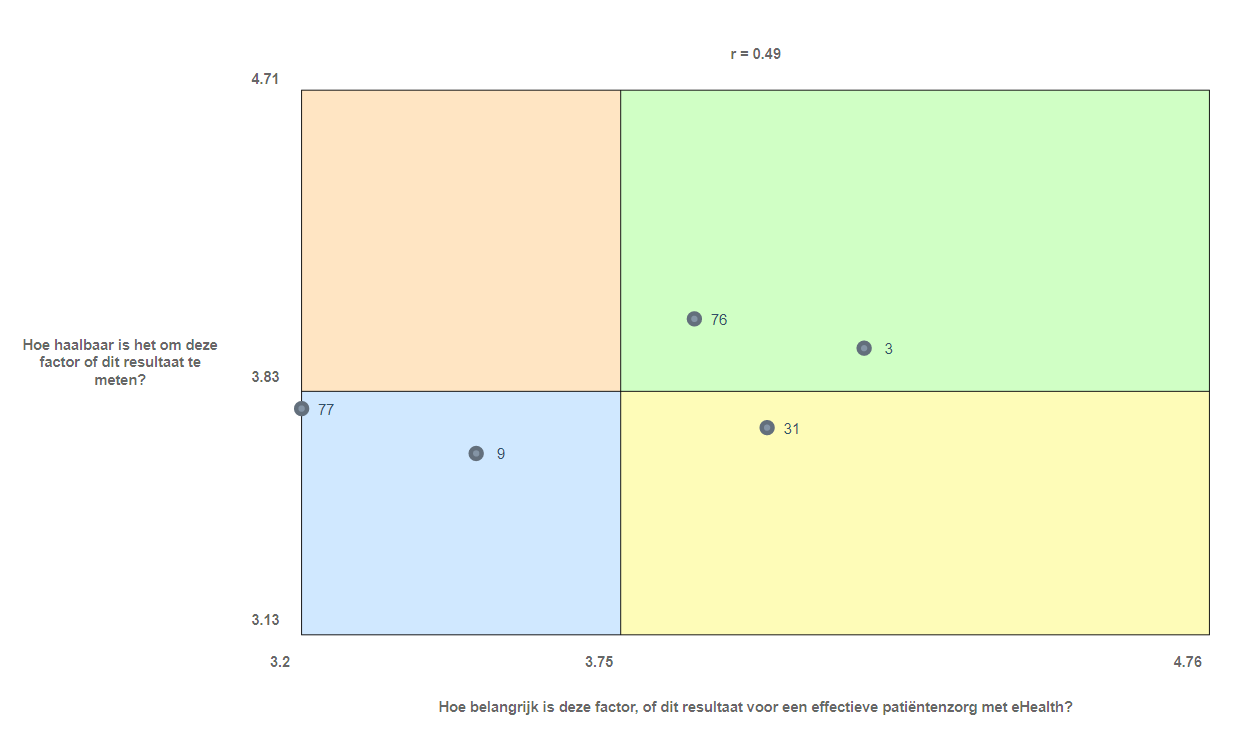


**Figure 8**. Go-zone cluster 8. Organization outcomes.

#### Cluster 9. End results for the patient

| **Description** | | **Rating scores** | | **Quadrant 78 factors** |
| --- | --- | --- | --- | --- |
| Outcomes for the patients; health, added value, satisfaction, ownership, convenience | | Importance | Measurability |  |
| **Nr.** | **Factor** | **Mean (SD)** | **Mean (SD)** |  |
| 75 | eHealth has added value for the patient. | 4.76 (0.4) | 4.18 (0.8) |  |
| 68 | The patient is satisfied. | 4.42 (0.6) | 4.40(0.6) |  |
| 65 | Treatment with eHealth contributes to the patient's self-reliance. | 4.28 (0.7) | 4.17 (0.9) |  |
| 66 | Improved patient quality of life. | 4.39 (0.6) | 3.92 (0.9) |  |
| 33 | The patient can integrate the use of eHealth in his or her daily life. | 4.32 (0.7) | 4.09 (0.8) |  |
| 64 | Treatment with eHealth has a positive influence on the patient's health. | 4.32 (0.7) | 4.04 (0.8) |  |
| 73 | eHealth provides logistical convenience for the patient. | 4.22 (0.7) | 4.25 (0.8) |  |
| 71 | The patient has easy access to care. | 4.18 (0.6) | 4.17 (0.8) |  |
| 69 | The patient is satisfied with the knowledge and skills of the health care professional. | 3.95 (0.6) | 4.00 (0.7) |  |
| 53 | Patient is therapy compliant. | 3.48 (1.0) | 3.75 (0.9) |  |


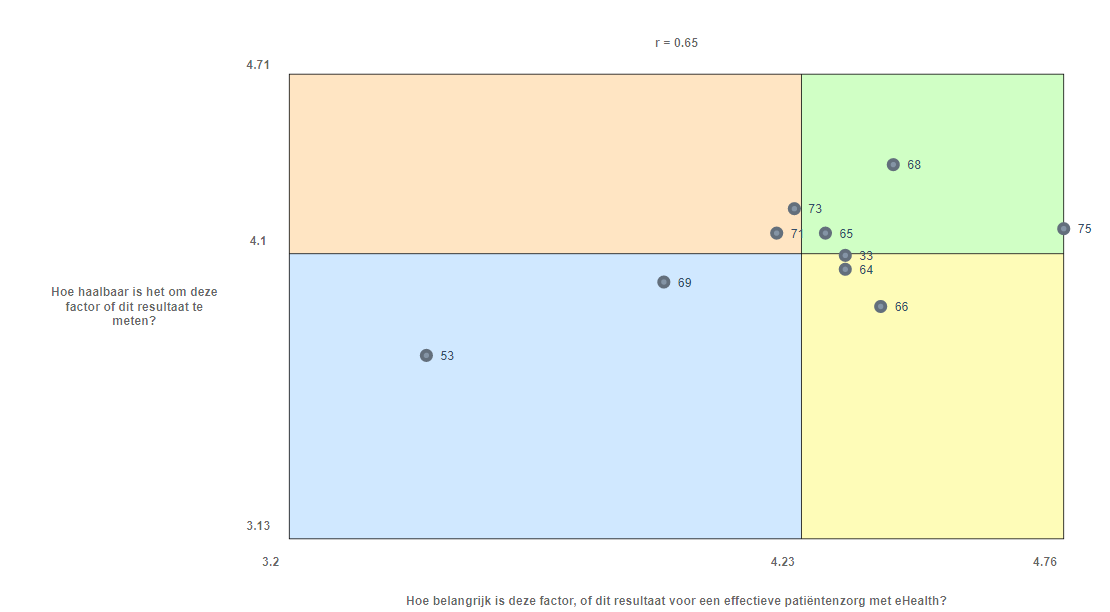


**Figure 9**. Go-zone cluster 9. End results for the patient.
